# Quantitative analysis of the effects of morphological changes on extracellular electron transfer rates in cyanobacteria

**DOI:** 10.1186/s13068-020-01788-8

**Published:** 2020-08-26

**Authors:** Tonny I. Okedi, Adrian C. Fisher, Kamran Yunus

**Affiliations:** 1 Department of Chemical Engineering and Biotechnology, University of Cambridge, Phillipa Fawcett Drive, Cambridge, CB3 0AS UK; 2 Cambridge Center for Advanced Research and Education in Singapore (CARES), 1 Create Way, #05-05 CREATE Tower, Singapore, 138602 Singapore

**Keywords:** Morphology, Extracellular electron transfer, Cyanobacteria, *Synechococcus elongatus* sp. PCC7942, Mass transfer

## Abstract

**Background:**

Understanding the extracellular electron transport pathways in cyanobacteria is a major factor towards developing biophotovoltaics. Stressing cyanobacteria cells environmentally and then probing changes in physiology or metabolism following a significant change in electron transfer rates is a common approach for investigating the electron path from cell to electrode. However, such studies have not explored how the cells’ concurrent morphological adaptations to the applied stresses affect electron transfer rates. In this paper, we establish a ratio to quantify this effect in mediated systems and apply it to *Synechococcus elongatus* sp. PCC7942 cells grown under different nutritional regimes.

**Results:**

The results provide evidence that wider and longer cells with larger surface areas have faster mediated electron transfer rates. For rod-shaped cells, increase in cell area as a result of cell elongation more than compensates for the associated decline in mass transfer coefficients, resulting in faster electron transfer. In addition, the results demonstrate that the extent to which morphological adaptations account for the changes in electron transfer rates changes over the bacterial growth cycle, such that investigations probing physiological and metabolic changes are meaningful only at certain time periods.

**Conclusion:**

A simple ratio for quantitatively evaluating the effects of cell morphology adaptations on electron transfer rates has been defined. Furthermore, the study points to engineering cell shape, either via environmental conditioning or genetic engineering, as a potential strategy for improving the performance of biophotovoltaic devices.

## Background

Biophotovoltaics (BPVs) promise a low-cost sustainable pathway for wastewater bioremediation with on-demand electricity or chemicals production. This is achieved by employing exoelectrogenic photosynthetic microorganisms requiring solar radiation and nutrients such as nitrates and phosphates that can be derived from wastewater. Electrical current generation in cellular BPVs has been demonstrated using various oxygenic photosynthetic microorganisms including eukaryotic microalgae such as *Chlorella vulgaris* [1, 2] and *Chlamydomonas reinhardtii* [3–5] as well as marine and fresh water species of prokaryotic cyanobacteria such as *Arthrospira maxima* [6], *Synechocystis* sp. PCC6803 [7–16] and *Synechococcus* sp. PCC7942 [17–19]. Sustainable technologies that integrate into current wastewater treatment systems are vital for meeting the policy demands of global climate change objectives. Key to delivering BPVs is understanding the electron transfer from the microorganisms to electrodes. This remains a major limiting factor for device performance. Two dominant schools of thought exist for the final transfer of electrons from the cyanobacteria cell membrane to electrodes which can be categorised as mediated (via an electron shuttle) or unmediated (direct) transfer. Furthermore, there is experimental evidence of both mechanisms occurring simultaneously [16].

**Fig. 1:**
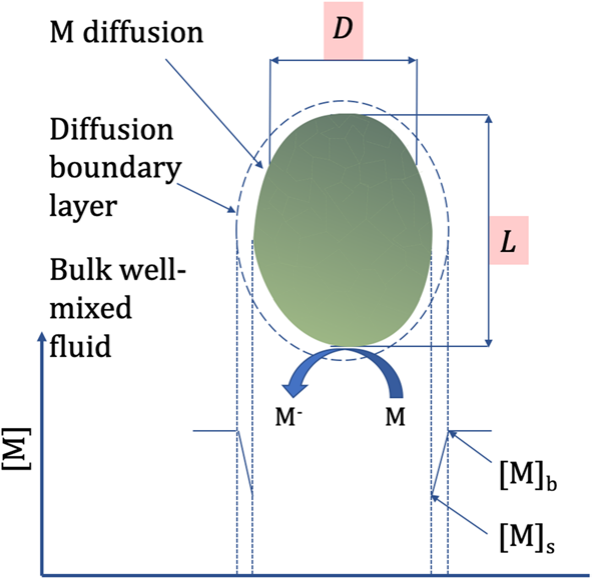
Schematic of exogenous or endogenously mediated extracellular electron transport. Mediators (M) diffuse through a boundary layer from the well-mixed bulk fluid to the cell surface of the cyanobacterium with projected length* L* and width* D*

Putative unmediated pathways found in literature are (1) an outer membrane c-type cytochrome (Omc) in direct contact with the anode; and (2) electrically conductive extracellular appendages (nanowires) that extend beyond the cell outer membrane in direct contact with the anode [13, 20, 21]. Mediated electron transport (Fig. 1) has been proposed to be via an unknown endogenous electron mediator that is released and then oxidised at the anode [15]. The mediator may or may not undergo redox cycling by re-entering the cell where it is reduced and then rereleased. In addition, non-lipid-soluble exogenous electron mediators (EEMs) such as ferricyanide (${[\hbox {Fe}(\hbox {CN})_6]^{3-}}$) have been used to achieve higher power densities in BPVs. These mediators can penetrate the outer membrane into the periplasmic space, but cannot cross the plasma membrane into the cytoplasm, and thus intercept electrons in the cell’s periplasmic region or outer membrane [1, 14]. Mediated electron transport introduces mass transfer phenomena to BPV systems which are known to limit the power output of electrochemical power devices such as fuel cells and batteries.

Stressing cyanobacteria cells by varying environmental conditions such as nutrient availability could be a way researchers employ to gain insight into the extracellular electron transport (EET) mechanisms in exoelectrogenic microorganisms. Notably, three studies have taken this approach: (1) starving *Synechococcus elongatus* sp. PCC7942 from iron increase EET rates; (2) feeding *Synechocystis *sp. PCC6803 with glucose, a carbon-fixed carbohydrate, increases EET rates; and (3) growing fresh-water *Synechocystis* sp. PCC6803 in high-salt medium increase EET rates [10, 15, 19]. However, understanding of the reasons why remains poor. Most discussions have been speculative, with a focus on linking the observed changes in EET rates to changes in the photosynthetic and/or respiratory machinery or expression of redox proteins and ion channels on cell membranes in response to the environmental conditions.

One area that has been largely unexplored in BPV research is how the morphological changes known to occur to cells in response to environmental stimuli affect the mass transfer properties of the system and, therefore, the overall EET rates. In addition to changing their physiology, metabolism, and biochemistry, cyanobacteria also modify morphology to adapt to changing environments, cope with stresses, and maximise utilisation of available resources [22]. Environmental factors known to affect cyanobacteria cell morphology include availability of nutrients (carbon, phosphorus, nitrogen and iron), light quality and colour, and stresses (temperature, oxidative, osmotic and pH) [22–24]. The shape of prokaryotic cells is thought to be driven predominantly by the need to increase diffusional efficiency. Diffusion is the fundamental mechanism by which prokaryotic cells bring the nutrients they need for sustenance to their cell surfaces as well as move nutrients and macromolecules intracellularly within their cytoplasms [24]. In particular, prokaryotic cells adapt to maximise their available surface-to-volume ratio. By doing so, the cells maximise the available surface area into which they can insert nutrient transporters, while minimising the volume of cytoplasm that each transporter must supply [24]. Thus, cells in low-nutrient environments typically have higher surface-to-volume ratios compared to cells with ready access to nutrients. For prokaryotes that obtain nutrients by direct contact with a solid body (e.g. cells growing in a biofilm rather than in suspension), it has been shown that cells filament (elongate) to increase the cell surface area in direct contact with the solid for faster nutrient uptake [24, 25].

Theoretically then, it appears that there is a synergistic relationship between morphological adaptation of cells to stressing conditions, and maximisation of electron transfer from cell to electrodes in BPV systems, either by diffusion or direct contact. However, without understanding the effects of morphological adaptations, it becomes difficult to discern to what extent measured changes in EET rates following exposure to environmental stimuli are attributable to physiological or metabolic changes occurring within the EET pathway, versus attributable to changes in morphology dependent mass transfer properties affecting extra- or intra-cellular diffusion rates. This limits our progress in understanding the complex EET pathways in cyanobacteria.

In this work, a novel method for quantifying the contribution of changes in morphology to the observed divergence in EET rates following exposure to environmental stimuli is developed. The method is demonstrated by quantifying to what extent morphological differences account for deviations in EET rates observed between *Synechococcus elongatus* sp. PCC7942 (PCC7942 hence forth) cells from nutritionally replete and nutritionally limited cultures.

### Overview of proposed method

Ferricyanide (${[\hbox {Fe}(\hbox {CN})_{6}]^{3-}}$) reduction rate is a common measure for the EET capacity of cyanobacteria. The molecule cannot penetrate lipid-membranes thus cyanobacteria reduce ferricyanide either on the outer membrane or in the periplasmic space after the molecule crosses the outer membrane [19, 26]. While the site of reduction of ferricyanide in cyanobacteria is still unknown, recent cyclic voltammetry work on iron-starved PCC7942 biofilms presented evidence of redox active proteins at the outer membrane level [19]. Furthermore, ferricyanide reduction rates are observed to increase when redox proteins are genetically engineered into the outer membrane of PCC7942 cells [27]. Based on these studies, the proposed method is developed with the assumption that the reduction site is on the outer membrane. The approach may still be valid when the reduction site is thought to be at the plasma membrane if it is reasonable to assume that the rate at which ferricyanide crosses the outer membrane into the periplasmic space is significantly faster than the rate of transport of the molecule from the bulk liquid to the outer membrane surface. If this cannot be assumed, then internal diffusion restrictions apply, and a more complex analysis is required which is not covered in this paper.

Ferricyanide assays take advantage of the fact that once reduced to ferrocyanide (${[\hbox {Fe}(\hbox {CN})_{6}]^{4-}}$), the molecule does not release free $\hbox {Fe(II)}$ which can be assimilated by the cells [2]. This allows the total reduction rate to be quantified by following the disappearance of ${[\hbox {Fe}(\hbox {CN})_{6}]^{3-}}$ or formation of ${[\hbox {Fe}(\hbox {CN})_{6}]^{4-}}$ spectrophotometrically [2].

In addition, the reduction reaction is mass transfer limited when there is an excess of cells for a given concentration of ferricyanide (see Ferricyanide assay in Methods for a review of reported mass transfer-limiting combinations of cell and ferricyanide concentrations for two species of cyanobacteria and algae) [28]. In mass transfer-controlled heterogenous catalytic surface reactions, the reaction rate is equivalent to the external mass transfer rate to the catalytic surface [29]. Under these conditions, the ferricyanide reduction rate (*r*) can be described using Fick’s law of diffusion, Eq. 1:

1 $$\begin{aligned} r=k\cdot A\cdot ([{\hbox {Fe}^{3+}}]_{{\text{b}}}-[{\hbox {Fe}^{3+}}]_{{\text{s}}}) \end{aligned},$$

where *k* is the mass transfer coefficient, *A* is the geometric outer membrane or plasma membrane area as appropriate, and $[{\hbox {Fe}^{3+}}]_{{\text{b}}}$ and $[{\hbox {Fe}^{3+}}]_{{\text{s}}}$ are the bulk and cell surface concentrations of ferricyanide, respectively. Higher reaction rates can be achieved by improving mass transport of the reactant between the cell surface and bulk solution [29].

Consider two cell cultures exposed to different environmental stimuli. Equation 1 enables differences in the morphology dependent properties (*k* and *A*) of cells from the two cultures to be quantitatively compared against differences in their EET capacity (*r*). This quantitative analysis may yield insights into the contribution of environmentally induced morphological changes to the measured deviations in mediated EET capacity.

To obtain the data necessary to perform the quantitative analysis, the following algorithm is proposed:

1. Identify from the literature or experimentation the cell and ferricyanide concentration combination for mass transfer limiting ferricyanide reduction.
2. Image cells using confocal microscopy to obtain projected cell dimensions.
3. Perform ferricyanide assays under mass transfer limiting conditions to estimate cell EET capacity.
4. Use a suitable stereological model to evaluate relevant 2D and 3D properties, namely the geometric cell surface area and cell volume, from the projected cell dimensions.
5. Use a suitable mass transfer correlation from literature to evaluate the mass transfer coefficient.
6. Evaluate the mediator concentration difference between the bulk fluid and the cell surface $([{\hbox {Fe}^{3+}}]_{{\text{b}}}-[{\hbox {Fe}^{3+}}]_{{\text{s}}})$ using Eq. 1.

## Results

### Characterisation of cultures

#### Growth rates

Cell concentration was measured every two days and the Gompertz bacterial growth model fit to the concentration profiles to estimate the specific growth rate ($\mu _{\text{max}}$), lag time ($\lambda$) and the maximal log of the relative cell number ($A=ln\left[ N_{\infty }/N_0\right]$) [30]. See Fig. S1, Additional file 1 for growth curves. The estimated values of $\lambda$ have large uncertainties for all growth conditions studied. This is because lag times are in the order of hours, whilst measurements were taken at a frequency of every 1–2 days, thereby losing the resolution required for more precise $\lambda$ estimates. Following the lag phase, the control cultures were in exponential growth until approximately day 11, with a $\mu _{\text{max}}$ of $0.55 \pm 0.06\,{{\text{day}}^{-1}}$ (doubling time of $13$ h) for the first 3 days under continuous light, and a $\mu _{\text{max}}$ of $0.20\pm 0.03\,{{\text{day}}^{-1}}$ (doubling time of $36$ h) following a change to a 12:12 light–dark cycle. The control cultures then entered the decline phase for the rest of the experiment. In comparison, PCC7942 cells grown under continuous light with an intensity of $70$ μmol m^−2^ s^−1^ (3x the light intensity used in this study) have a reported doubling time of $8.7\,\pm\,0.7$ h [31]. After the lag phase, the continuously diluted cultures were maintained in exponential growth for the remainder of the experiment at an average $\mu$ of $0.36\pm 0.01$ day^−1^ (average doubling time of $20$ h) following the change to a 12:12 light–dark cycle. An average doubling time of $10.3$ h has been reported for continuously diluted PCC7942 cultures under continuous light of intensity $125$ μmol m^−2^ s^−1^ [32].

#### Projected cell dimensions and stereological properties

**Fig. 2:**
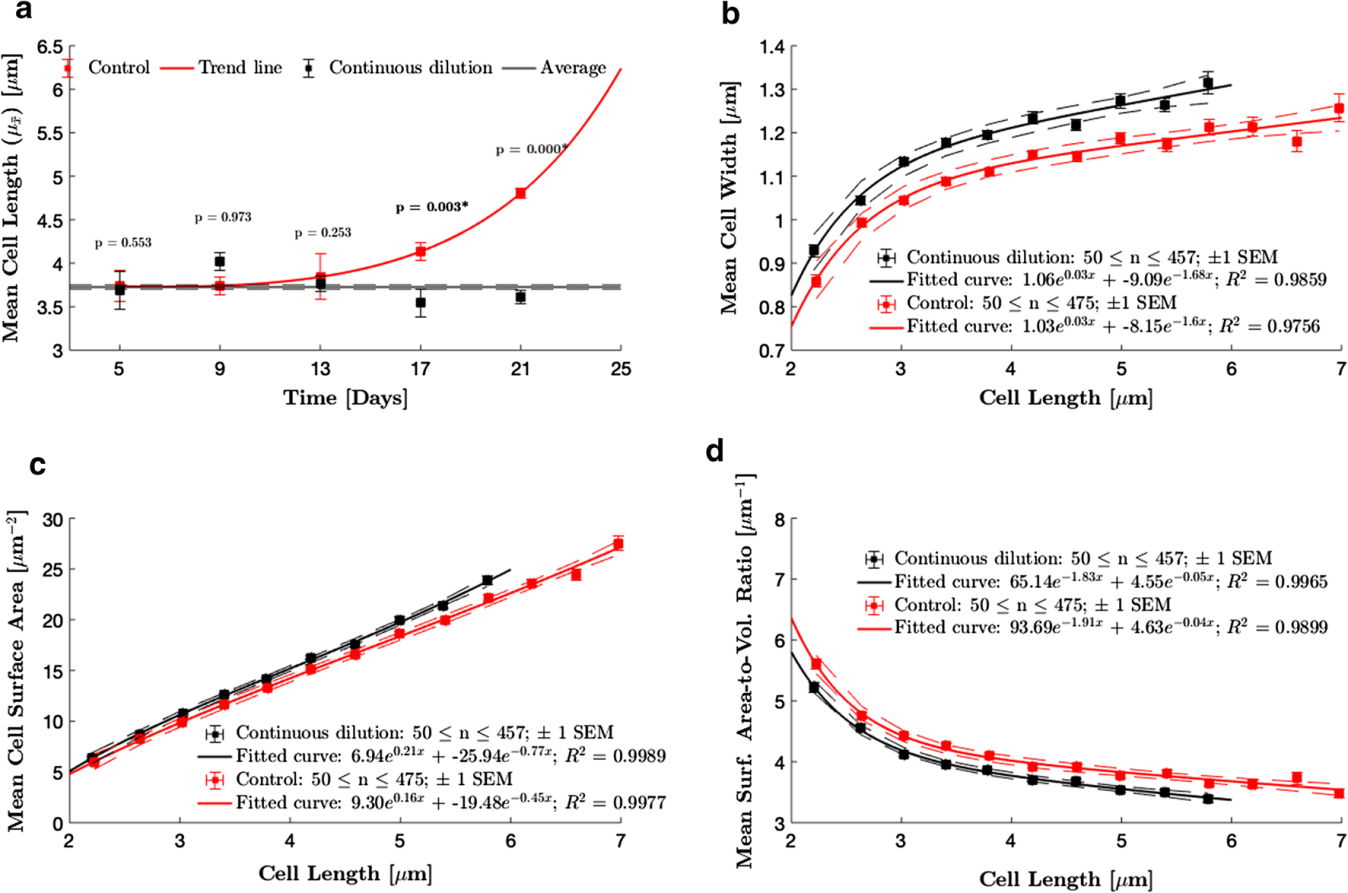
Projected dimensions and stereological properties. **a** Mean cell length (mean of sampling distribution of the mean, $\mu _{\bar{x}}$) profiles. Error bars show standard deviation of the sampling distribution of the mean. Means and standard deviations are weighted by sample size. A two-tailed Student’s *t* test at 5% significance was used for pairwise comparison of cell lengths. Significant p-values (above data points) are asterisked. The red trend line is to guide the eye and is not a regression model. The black trend line and black dotted lines show the average cell length over the duration of the experiment for the cells in the continuously diluted cultures $\pm\, 1$ SEM, respectively. **b** PCC7942 cell width vs. cell length. **c** PCC7942 cell area vs. cell length. **d** PCC7942 cell surface-to-volume ratio vs. cell length. Plots **b**, **c** and **d** were plotted using data collected over the duration of the experiment from 2973 and 3636 cells from the continuously diluted and control cultures, respectively. The data were discretised by cell length into bins of 0.4 μm wide. Mean values were calculated from bins with at least 50 data points to produce the plot. Solid lines are regression models and dotted lines are 95$\%$ confidence bounds

Cell lengths and widths were probed using confocal imagery every 4 days prior to conducting ferricyanide assays (see Figs. S2–S5, Additional file 1 for example confocal images obtained). Figure 2a shows the mean length profiles of cells in the two growth conditions (width profiles not shown). Histograms of the cell length distributions from day 1 onwards can be found in Fig. S6, Additional file 1 for both growth conditions. Histograms from day 1 indicate that as cells exited the lag phase, their morphology was more representative of conditions in the stock culture rather than cells in their new environments. Both cultures exhibited elongated cells with mean lengths of about 5.7 μm, indicating adaptation to the nutrient-depleted stationary phase stock culture. Upon entering the exponential phase, mean cell length for the continuously diluted cultures fluctuated about an average of 3.7 ± 0.3 μm for the remainder of the experiment. The fluctuations are attributed to batch-to-batch variation during sampling for imaging rather than a statistically significant difference in cell length. Control cultures in the exponential phase of growth (days 5 and 9) had the same mean cell length as the continuously diluted cultures within experimental uncertainty, but cells began to elongate when the cultures transitioned to the decline phase (day 11 onwards). The cells elongated by approximately $1.0\pm 0.3$ μm on average between days 13 and 21.

**Fig. 3:**
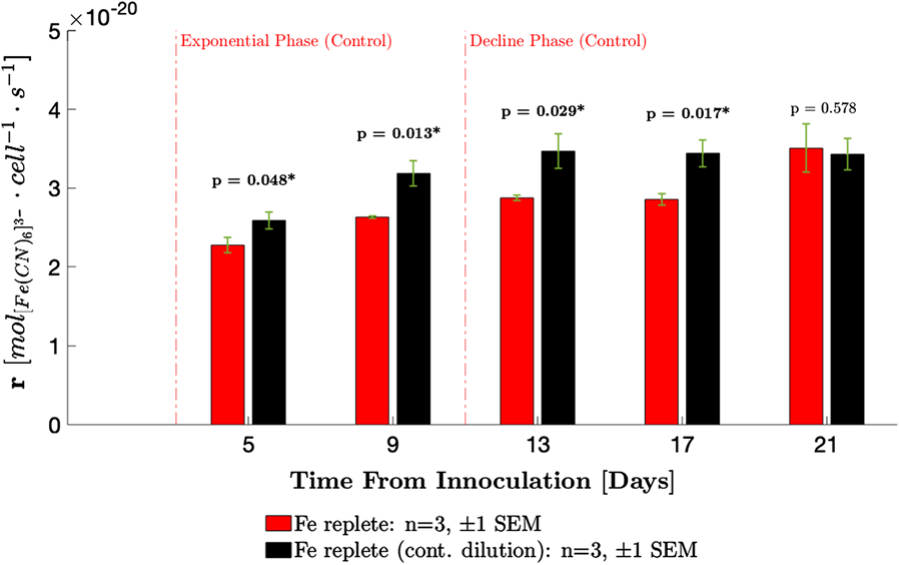
Ferricyanide reduction rates. Error bars show ± 1 SEM. To assess significance of the pairwise difference in rates, a one-tailed Student’s t test at 5% significance level was conducted, with alternative hypothesis that reduction rates are higher for the continuously diluted cultures. Significant* p* values are asterisked (above bars)

All the cell length and width data collected over the 21-day period were further analysed to develop regression models relating cell length to cell width (Fig. 2b), cell geometric surface area (Fig. 2c), and cell surface-to-volume ratio (Fig. 2d). The latter two properties were evaluated for each cell using a rod-shaped model of the cyanobacterium (Eqs. 5 and 6 in Methods). Data of 2973 and 3636 cells from the continuously diluted and control cultures, respectively were used to generate the regression curves. The regression models were then used to estimate the mean cell area (Fig. 3b) and mean surface-to-volume ratios (Fig. 3c) from the cell lengths measured prior to each ferricyanide assay. The advantage of this approach is to reduce the error in the estimate of the population means for each property.

Under both growth conditions, cell width was found to increase with cell length. However, for cells in the approximately 3–7 μm range, increase in cell width was found to be minimal ($\le 0.2$ μm), corroborating that PCC7942 cells increase volume mainly by increasing pole-to-pole length [33]. In addition, this confirms that cell length should be used as the characteristic dimension for the evaluation of the mass transfer coefficient. Cells in the nutritionally replete continuously diluted cultures were 8–9% wider. As a result, the cells had larger surface areas but smaller surface-to-volume ratios at all cell lengths.

### Quantifying the effect of morphological changes on EET rates

#### Ferricyanide reduction rates (r)

Ferricyanide assays were conducted every 4 days upon the cultures entering the exponential phase of growth, when cells had fully acclimated to their new environment. For the continuously diluted cultures, ferricyanide reduction rates increased gradually before reaching an apparent plateau of about $3.5\cdot 10^{-20}\,$ mol cell^−1^ s^−1^ on day 13 and were consistently higher vs. control on days 5, 9, 13 and 17 (Fig. 3). Reduction rates in the control cultures increased more slowly over the duration of the experiment, reaching the same level as that of continuously diluted cultures by day 21. The maximum reduction rates recorded are an order of magnitude lower than those of the highly exoelectrogenic microalgae *Chlorella vulgaris*, which has a reported mass transport limited reduction rate of $8.3\cdot 10^{-19}\,$ mol cell^−1^ s^−1^ in the dark (converted from $3$ nmol (10^6^ cells^−1^) h^−1^) using $1$ mM ferricyanide and $1.4\cdot 10^{8}\,$ cells ml^−1^ [2].

**Fig. 4:**
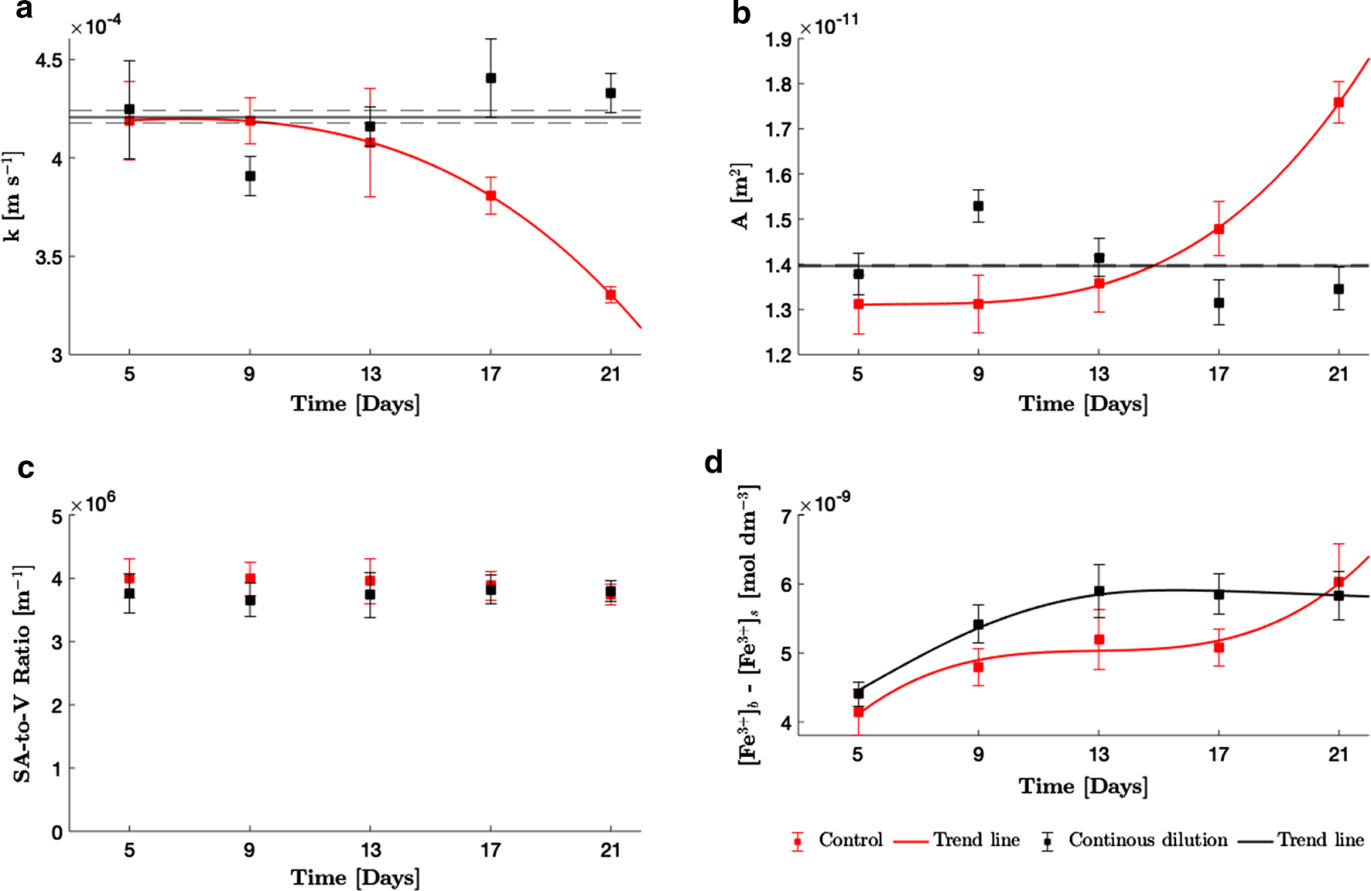
Profiles of mass transfer properties. **a** Mean mass transfer coefficient. Error bars are calculated by combining errors using error propagation equations. **b** Mean geometric cell surface area. For **a** and **b**, the red trend lines are to guide the eye and are not regression models. The black trend line and black dotted lines show the average value of the properties ± 1 SEM, respectively, over the duration of the experiment for the cells in continuously diluted culture. Fluctuations about this average are attributed to batch-to-batch error in cell length measurements. **c** Mean cell surface-to-volume ratio. Area and surface-to-volume ratios for each day were predicted from the regression curves in Fig. 2c, d, respectively, using the length data in Fig. 2a. Error bars show the difference between the predictions obtained from the regression curves and the 95% confidence bounds. **d** Estimated mean ferricyanide concentration difference between bulk medium and cell surface. Error bars are calculated by combining errors in r, k and A using error propagation equations. Both black and red trend lines are to guide the eye and are not regression models

The reduction rates were also normalised by chlorophyll *a* content (see Fig. S7, Additional file 1) to compare to values reported in literature for PCC7942. The reduction rates for the control culture increased from $73.5\,{\text{pM}}\,{\text{nM}}_{{{\text{ChlA}}}}^{{ - {\text{1}}}} \,{\text{min}}^{{ - 1}}$ pM nM_ChlA_^−1^ min^−1^ on day 5 to $96\,{\text{pM}}\,{\text{nM}}_{{{\text{ChlA}}}}^{{ - {\text{1}}}} \,{\text{min}}^{{ - 1}}$ on day 13. Comparatively, reduction rates of 50 and $27\,{\text{pM}}\,{\text{nM}}_{{{\text{ChlA}}}}^{{ - {\text{1}}}} \,{\text{min}}^{{ - 1}}$ are reported for assays conducted at the same cell and ferricyanide concentration but a light intensity of $90$  μmol m^−2^ s^−1^ after 2 and 14 days of culturing, respectively [19]. An average ferricyanide reduction rate of $15\, {\text{pM}}\;{\text{nM}}_{{{\text{ChlA}}}}^{{ - 1}} \;{\text{min}}^{{ - {\text{1}}}}$ (converted from $0.9\, {\text{mM}}\;{\text{mM}}_{{{\text{ChlA}}}}^{{ - 1}} \;{\text{h}}^{{ - 1}}$) is reported using 30$\%$ of the cell concentration used in this study and a light intensity of $80$ μmol m^−2^ s^−1^ [27]. It should be noted that normalising ferricyanide reduction rates by chlorophyll *a* content has two key issues that make direct comparisons difficult. First, there is an inherent assumption that the photosynthetic light reactions limit the overall reduction reaction. However, when the reaction is kinetically limited, it is known that the kinetics of electron transport to the cell surface are the bottleneck [7]. The conditions in this study are such that mass transfer is limiting, adding further complexity. Second, chlorophyll *a* content can change significantly with media and light conditions. Even if the same cell concentration is used for each ferricyanide assay, the chlorophyll *a* content per cell may differ from day to day, resulting in misleading comparisons. In example, the reduction rate normalised by cell number in Fig. 3 shows a clear increase from day 17 to day 21 for the control cells; however, the reduction rate appears not to change within experimental error when normalised by chlorophyll *a*. This is because there is an increase in the chlorophyll *a* content per cell during this time (see chlorophyll *a* profile in Fig. S8, Additional file 1).

#### Mass transfer coefficient (k)

Equation 4 (see Methods) was used to evaluate the mass transfer coefficients using the projected cell lengths reported in Fig. 2a and parameters shown in Table 1. For the diffusivity of ferricyanide, the average value from three studies, $7.504\cdot 10^{-10}$, $7.36\cdot 10^{-10}$ and $7.26\cdot 10^{-10}\,{\text{m}}^{2}\, {\text{s}}^{-1}$ at $31.2$ °C, $30.3$ °C and $25$ °C, respectively, was used [34–36]. This is to account for temperature fluctuations during the ferricyanide assays, particularly temperature drops as the incubator was opened to the $20$ °C ambient room temperature to take aliquots, and temperature overshoots above set-point when the incubator was closed. It was assumed that the BG11 medium has the same viscosity and density as pure water because salt concentrations are dilute.

**Table 1:** Parameters used to evaluate mass transfer coefficient

Parameter	Unit	Value	References
$D_{AB}$ (at 25–31 °C)	m^2^ s^−1^	$7.44\, 10^{-10}$	[34–36]
$\rho _{{\text{p}}}$	kg m^−3^	1040	[48]
$\rho _{{\text{c}}}$ (at 30 °C)	kg m^−3^	995.65	[49]
*g*	m s^−2^	9.80665	[42]
${\mu _{\mathrm{C}}}$ (at $30$ °C)	kg m^−1^ s^−1^	$7.9735\, 10^{-4}$	[49]

It was demonstrated that cells in continuously diluted cultures remained roughly the same length throughout the experiment, while cells in the control culture elongated upon entry into the decline phase. Analogously, *k* in the continuously diluted cultures remained relatively constant, fluctuating about an average, while the control cultures experienced a −20.5$\%$ reduction in *k* (Fig. 4a) as cells elongated by 28.6$\%$ over the same period (Fig. 2a).

#### Cell surface area (A) and surface-to-volume ratio

The cell area profiles over the duration of the experiment are shown in Fig. 4b. Mean geometric cell surface area for the continuously diluted cultures fluctuates about an average of $14\pm 0.84$ μm^2^ over the duration of the experiment. The fluctuations are due to batch-to-batch error in measurements of cell length. On average, the control cells experienced a 34$\%$ increase in geometric surface area over the duration of the experiment as the cells elongated over time. Control cultures experienced a marginal decrease in the mean surface-to-volume ratio (− 4.8$\%$) over the duration of the experiment (Fig. 4c). However, the decrease cannot be said to be significant within experimental uncertainty.

#### Estimation of mediator concentration difference between bulk medium and cell surface $([\hbox {Fe}^{3+}]_{{\text{b}}}-[\hbox {Fe}^{3+}]_{{\text{s}}})$

Once the reduction rates, mass transfer coefficient and geometric surface area were known, the average ferricyanide concentration differences between the bulk medium and the cell surface were evaluated using Eq. 1 (Fig. 4d). This is an important metric since concentration gradients are the driving force for diffusion. It was expected that since the reduction reaction is mass transfer limited, the value of $[{\hbox {Fe}^{3+}}]_{{\text{s}}}$ should be zero at all times, resulting in a value of $([{\text {Fe}^{3+}}]_{{\text{b}}}-[{\text {Fe}^{3+}}]_{{\text{s}}})$ that remains largely constant over time for both growth conditions. The observed increase in $([{\hbox {Fe}^{3+}}]_{{\text{b}}}-[{\hbox {Fe}^{3+}}]_{{\text{s}}})$ for the continuously diluted cultures from day 5 to 13 was, therefore, surprising. A similarly unexpected rise in $([{\hbox {Fe}^{3+}}]_{{\text{b}}}-[{\hbox {Fe}^{3+}}]_{{\text{s}}})$ was observed for the control cultures. Estimated $([{\hbox {Fe}^{3+}}]_{{\text{b}}}-[{\hbox {Fe}^{3+}}]_{{\text{s}}})$ values were correlated with the cells’ morphological properties, namely cell length, width, geometric surface area, and surface-to-volume ratio, as well as a theoretical media iron concentration profile (see Figs. S9 and S10, Additional file 1 for correlation plots and calculated iron concentration profile, respectively). No strong correlations to the morphological properties were seen in either the control or continuously diluted cultures. A strong negative correlation to the iron concentration was seen for both growth conditions.

#### Quantifying the effect of morphological changes on EET rates

With all variables in Eq. 1 known, the effect of changes in *L*, the characteristic morphological dimension, on EET rates was quantified. Given no correlations were found between morphology-dependent properties and $([{\hbox {Fe}^{3+}}]_{{\text{b}}}-[{\hbox {Fe}^{3+}}]_{{\text{s}}})$, it was concluded that changes in *L* affect reduction rates predominantly through *k* and *A*. Increasing *L* has two opposing effects on ferricyanide reduction rates. That is, cell elongation increases *A* but decreases *k* (Eqs. 4 and 5 in Methods). The chain rule can be used to decouple these two opposing effects on the ferricyanide reduction rate as shown in Eq. 2.

2 $$\begin{aligned} {{\frac{\partial r}{\partial L}}}=\left( \frac{\partial r}{\partial k}\cdot \frac{\partial k}{\partial L}\right) +\left( \frac{\partial r}{\partial A}\cdot \frac{\partial A}{\partial L}\right) =\left[ \left( A\cdot \frac{-2D_{{\text{AB}}}}{L^{2}}\right) +\left( k\cdot \pi D\right) \right] \cdot \left( [{\hbox {Fe}^{3+}}]_{{\text{b}}}-[{\hbox {Fe}^{3+}}]_{{\text{s}}}\right) \end{aligned}.$$

**Fig. 5:**
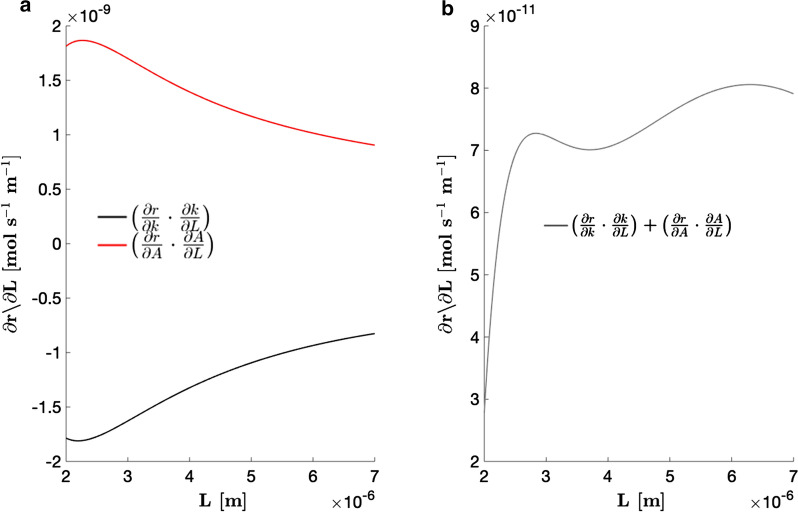
Derivative of *r* with respect to *L*. **a** Decoupled derivative showing rate of change in ferricyanide reduction rate as a result of changes in *k*(*L*) and *A*(*L*) for a unit mediator concentration difference between the bulk medium and cell surface, $\left( [\hbox {Fe}^{3+}]_{{\text{b}}}-[\hbox {Fe}^{3+}]_{{\text{s}}}\right) =1$mol m^−3^. ** b** Derivative of ferricyanide reduction rate with respect to cell length

Equation 2 was evaluated using the parameters in Table 1 and the dimension data obtained for cells in the control culture as presented above, for a unit mediator concentration difference between the bulk medium and cell surface, $\left( [\text {Fe}^{3+}]_{{\text{b}}}-[\text {Fe}^{3+}]_{{\text{s}}}\right) =1$ mol m^−3^. Results of this calculation (Fig. 5a, b) indicate that the rate of increase of *A* with cell length is substantially greater than the rate of decrease of *k* with cell length.

**Fig. 6:**
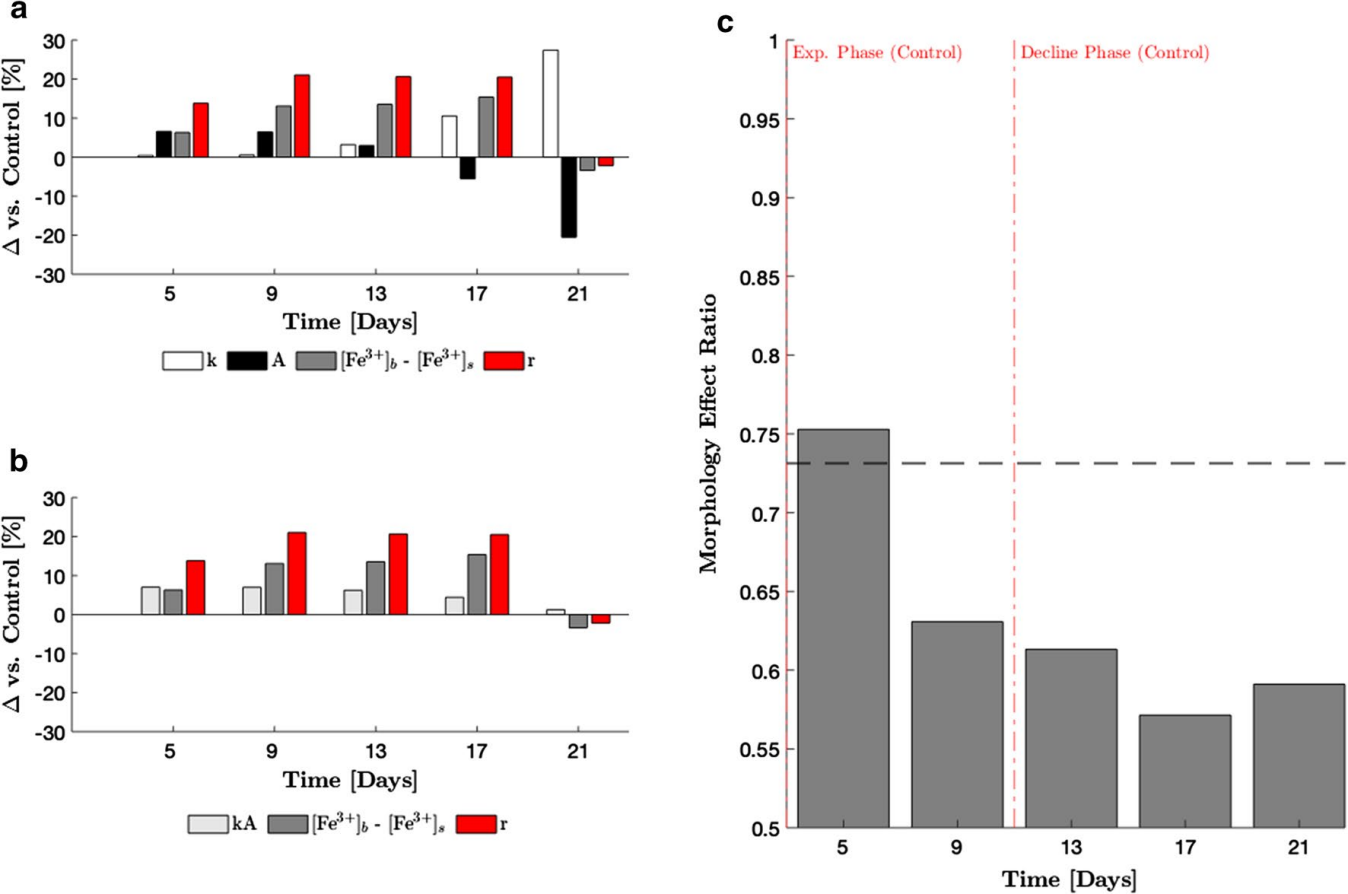
Quantitative analysis of morphological changes. **a** Fractional difference in each of the independent and dependent variables vs. control. **b** Fractional change in $k\cdot A$, $\left( [Fe^{3+}]_{b}-[Fe^{3+}]_{s}\right)$ and *r* vs. control. The fractional change in $k\cdot A$ shows the resultant effect of changes in morphology and was calculated from the following relationship: $\left( 1+\Delta \left( k\cdot A\right) \right) =(1+\Delta k)\cdot (1+\Delta A)$. **c** Morphology effect ratio applied to the observed changes in EET rates between the nutritionally replete cells in the continuously diluted culture and the relatively nutrient limited cell in the control. The black dotted line at MER = 0.7311 is the value of MER when morphological and unknown physiological/metabolic effects on EET rates are equal

The fractional difference ($\Delta$) in *k*, *A* and $([\text {Fe}^{3+}]_{{\text{b}}}-[\text {Fe}^{3+}]_{{\text{s}}})$ between the continuously diluted and control cultures were compared with the corresponding fractional differences in *r* (Fig. 6a). In addition, the fractional difference in the product of *k* and *A*, the morphology-dependent properties, were compared to $\Delta \left( [\hbox {Fe}^{3+}]_{{\text{b}}}-[\hbox {Fe}^{3+}]_{{\text{s}}}\right)$ and $\Delta r$ (Fig. 6b). The fractional differences are related by $(1+\Delta r)=(1+\Delta k)\cdot (1+\Delta A)\cdot (1+\Delta \left( [\hbox {Fe}^{3+}]_{{\text{b}}}-[\hbox {Fe}^{3+}]_{{\text{s}}}\right) ).$ Figure 6a, b enables visualisation of how changes in morphology (manifested as $\Delta k$ and $\Delta A$) are affecting mass transfer properties, and the resultant magnitude of these changes relative to the deviation in the EET rate.

To have a single quantitative measure of the effect of morphological changes on EET rates, the morphology effect ratio (MER) was defined. It is the ratio of the absolute value of $\Delta \left( k\cdot A\right)$ to the absolute value of $\Delta \left( [\hbox {Fe}^{3+}]_{{\text{b}}}-[\hbox {Fe}^{3+}]_{{\text{s}}}\right)$, transformed by the sigmoid function (Eq. 3). Transformation by the sigmoid function is done to constrict the value of MER between 0.5 and 1 for convenience and simpler interpretation of results. Without this transformation, MER values would be inconveniently high for very small $\left| \Delta \left( [\hbox {Fe}^{3+}]_{{\text{b}}}-[\hbox {Fe}^{3+}]_{{\text{s}}}\right) \right|$. An MER value of $\sim$0.7311 (ratio of absolute values equals 1) signifies that morphological changes and unknown physiological/metabolic changes equally contribute to the observed change in EET rates. Values of MER greater than $\sim$0.7311 signify that morphological changes dominate and vice versa for MER values below $\sim$0.7311.

3 $$\begin{aligned} {\text{MER}}=1/\left( 1+{\text{exp}}\left(-\frac{\left| \Delta \left( k\cdot A\right) \right| }{\left| \Delta \left( [\hbox {Fe}^{3+}]_{{\text{b}}}-[\hbox {Fe}^{3+}]_{{\text{s}}}\right) \right| }\right)\right) \end{aligned}$$

Figure 6c shows the MER concept applied to the results presented above. The MER was found to be above the 0.7311 threshold on day 5, and then decline over time until day 17. There was a small increase in the MER on day 21.

## Discussion

The morphological results resonate with the hypothesis that prokaryotic cells adapt cell shape predominantly to maximise intra- and extra-cellular diffusion of nutrients. The control culture cells in the exponential phase are thinner than cells in the continuously diluted cultures to increase their surface-to-volume ratio in a more nutritionally limited environment. As nutrients are depleted further and the control cultures enter into decline, the cells elongate. Elongation increases the cells’ solution facing external surface area for enhanced diffusion with little detriment to the surface-to-volume ratio. This avoids additional strain on membrane-bound nutrient transporters. Synergistically, this increase in the solution facing surface area is beneficial to the rate of diffusion of ferricyanide and ferrocyanide to and from the cell surface, respectively.

In this work, the applicability of the rod-shaped model was verified by comparing volumes calculated using the model, with volume measurements obtained using a Coulter counter. The values agreed within experimental uncertainty (see Fig. S11, Additional file 1), justifying the rod-shaped assumption. Particle counters using the Coulter principle are an established and accurate technology for rapidly obtaining information about cell size and volume. However, while particle volume readings output by such counters are independent of cell shape, the output particle diameters are typically the equivalent spherical diameter (ESD) [37]. The ESD is misrepresentative of the true length of the rod-shaped PCC7942 cells. Thus, confocal imaging with automated image processing is a superior alternative for obtaining cell dimension data for non-spherical cells.

Because the density difference between PCC7942 cells and BG11 media is small (Table 1), the mass transfer coefficient is predominantly determined by the first term on the right-hand side (RHS) of Eq. 4. For all values of *k* calculated, for both the control and continuously diluted cultures, the value of the first term on the RHS of Eq. 4 has an order of magnitude of $10^{-4}$, while the second term on the RHS is of the order $10^{-5}$. Small variations or fluctuations in cell density, either above or below that of BG11, are, therefore, of negligible consequence to the overall reduction reaction. It is the evolution of cell length that is of particular importance as it is the predominant determinant of changes in both *k* and *A* in Eq. 1.

Decoupling the derivative of *r* with respect to *L* showed that while the diffusional flux decreases as the cell elongates (negative derivative of *k* with respect to *L*), the concomitant increase in the surface area available to flux (positive derivate of *A* with respect to *L*) more than compensates for the decline. Thus, the net result of cell elongation is an increase in reduction rates for a given value of $([\hbox {Fe}^{3+}]_{{\text{b}}}-[\hbox {Fe}^{3+}]_{{\text{s}}})$. This is an important finding because it provides an explanation to the hitherto unattributed observation that PCC7942 ferricyanide reduction rates increase over time [19]. The results presented here suggest that this is partially due to increasing cell surface area as cells elongate in adaptation to the increasingly nutrient-limited environment in the decline and stationary phases of growth.

The unexpected rise in $([\hbox {Fe}^{3+}]_{{\text{b}}}-[\hbox {Fe}^{3+}]_{{\text{s}}})$ values with time suggests that the value of $[\hbox {Fe}^{3+}]_{{\text{s}}}$ is a finite, non-zero value that decreases with time (a constant $([\hbox {Fe}^{3+}]_{{\text{b}}}$ is used for all assays). One explanation for this is that ferricyanide ions are being adsorbed and trapped on the cell surface during the ferricyanide assays [2]. The amount of trapped ferricyanide ions may be decreasing as the cells undergo physiological and metabolic adaptations to nutrient depletion in the growth media. In particular, the strong negative correlations in both cultures between $([\hbox {Fe}^{3+}]_{{\text{b}}}-[\hbox {Fe}^{3+}]_{{\text{s}}})$ and the declining bioavailable iron concentration in the growth media suggests adaptations aimed at increasing iron uptake capacity through a reductive mechanism [19]. In an attempt to assimilate the trapped ferricyanide ions, the cells may be redirecting additional electrons to the surface, thereby reducing trapped ferricyanide to ferrocyanide, leading to a decrease in $[\hbox {Fe}^{3+}]_{{\text{s}}}$ over time.

Interestingly, the theoretical bioavailable iron concentration in the continuously diluted cultures flattens out by day 13, which coincides with a plateau in the estimated value of $([\hbox {Fe}^{3+}]_{{\text{b}}}-[\hbox {Fe}^{3+}]_{{\text{s}}})$. By this time, the media environment in these cultures becomes pseudo-constant. Variations in the concentrations of nutrients in the BG11 medium, as well as in light penetration, are kept to a minimum by the continuous dilution thus eliminating the need for further physiological or metabolic adaptations. Contrarily, in the control, the gradual depletion of nutrients as well as reduced light penetration as a result of self-shading by the growing population density of cells necessitates ongoing adaptation to the changing environment. The observed increase in the chlorophyll *a* content per cell from day 17 onwards and a higher pH from day 11 onwards were evidence of the evolving conditions (see Figs. S8 and S12, Additional file 1 for chlorophyll *a* and pH profiles). Additionally, dissolved oxygen measurements may provide further insights into the changing physiology of the cells.

A second explanation for the unexpected rise in $([\hbox {Fe}^{3+}]_{{\text{b}}}-[\hbox {Fe}^{3+}]_{{\text{s}}})$ is that there is a change in the zeta potential of the cells, which is a measure of the surface charge (in addition to a few layers of bound water) of the particles. The zeta potential of several species of cyanobacteria, namely *Tetraselmis suecica, Chlorococum* sp., *Chlorella* sp. XJ-445 and *Desmodesmus bijugatus* XJ-231 has been reported to become less electronegative as cultures transition from the exponential to the stationary phase [38, 39]. The zeta potential of the PCC7942 cells may be exhibiting similar behaviour. In the earlier days of growth, relatively high electronegativity of the cells may be repelling the negative ferricyanide ions as they approach the cell surface, thereby reducing the effective diffusivity of the ions. As the cells become less electronegative with time, repulsive forces are attenuated and ferricyanide ions can reach the cell surface at a faster rate. There is some visual evidence of changing zeta potential in the control cells from the raw confocal images (see Figs. S2–S4, Additional file 1). The cells appear further away from each other on day 5 than on days 17 and 21 suggesting stronger repulsive forces between cells on this day [38]. However, zeta potential measurement should be conducted to test this hypothesis in future work.

While both scenarios presented above point to on-going physiological or metabolic adaptations, it is probably safe to assume that these do not occur over the short 2-h timescale of the ferricyanide assays. This is because nutrient uptake is gradual and culture doubling times are at least 6 and up to 18 times longer. Therefore, the proposed approach is still capable of extracting the contribution of morphological differences on EET rates within a 2-h ferricyanide assay without significant influence from physiological changes.

The MER was defined and applied in this work. The MER shows that in the early exponential phase (day 5), the difference in EET rates between the nutritionally replete continuously diluted cultures and the relatively nutritionally limited control cultures is marginally dominated by morphological differences. This is largely attributable to the greater external facing surface area of the wider continuously diluted cells during this time. As the control cells elongate, the difference in surface areas is reduced and by day 17, reversed. Although the gains in surface area are counteracted by a concomitant decline in mass transfer coefficient, the resultant effect is still an increase in $k\cdot A$ over time. As such, $\Delta \left( k\cdot A\right)$ becomes smaller and physiologic or metabolic differences attributable to adaptations to the changing cell environment can be concluded to dominate the measured difference in EET capacity. This suggests that if additional measurements such as transcriptional analysis or fluorescence yield were conducted on the cultures to compare differences in physiology or metabolism, conclusions should be made on measurements taken on days 9, 13 and/or 17 only. On these days, there is a statistically significant departure in EET rates, *and* physiological/metabolic state differences likely domineer the measured difference in EET rates.

The MER is a simple ratio that provides researchers applying environmental conditioning to study the complex EET pathways in cyanobacteria with a quantitative tool to discriminate between environmental conditions that may give insights into pathways, from environmental conditions that result in changes in EET rates by simply changing the cell morphology and the associated mass transfer properties.

More generally, the results presented here provide further support for engineering cyanobacteria cell morphology, either through environmental conditioning or genetic means, for enhancing the performance of BPV systems. In particular, the finding that ferricyanide reduction rates increase with cell surface area over the growth cycle of the cultures should be investigated in BPV systems where suspended planktonic cells are used in the anodic chamber, and the electron transport to the anode is mediated by an endogenous or exogenous mediator. Engineering cell shape to maximise surface area may concomitantly enhance the counter transfer of reduced mediator to the anode, thereby delaying the onset of mass transfer losses to higher current densities. It should be noted that in addition to mass transport losses, electrochemical devices face various other losses such as activation and ohmic losses that would not be directly addressed by optimising cell morphology. As such, it is important to identify other key bottlenecks in the performance of a system to achieve the maximum benefits of this approach. Additionally, further work should investigate engineering cell morphology to enhance mass transport and direct electron transfer in systems where biofilms are used in the anode. This may include matching cell dimensions to electrode pore sizes and optimising cell shape to increase contact area between cells and the electrode surface.

## Conclusions

PCC7942 adapt cell morphology in accordance to their evolving external environment to maximise diffusional efficiency for nutrient uptake. These adaptations can be synergistic to optimal shapes for mediated and unmediated extracellular electron transport in BPV systems. In the case rod-shaped cells, wider or elongated cells were found to exhibit enhanced EET rates by increasing the solution facing geometric surface area available for the flux of the electron mediator. This important result reveals cell elongation and cell widening as potential strategies for enhancing EET rates in BPV systems catalysed by rod-shaped cells and mediated by a non-lipid-soluble exogenous mediator such as ferricyanide. More generally, engineering cyanobacteria morphology can be a strategy for improving the performance of BPV systems. The morphology effect ratio was defined and an algorithm for obtaining the necessary quantitative information to evaluate the ratio delineated. The simple ratio provides researchers with an easy-to-interpret value denoting to what extent morphological changes may account for the observed variations in EET rates. It is envisioned the ratio will aid researchers identify the most insightful environmental stimuli and time periods during the bacterial growth cycle for probing EET pathways in cyanobacteria.

## Methods

### Cyanobacteria culturing conditions

A stock culture of the cyanobacterium *Synechococcus elongatus* sp. PCC7942 (Pasteur Culture Collection), PCC7942 henceforth, was grown in liquid blue-green medium (BG11) [40]. The stock culture was grown in air at room temperature (approximately $20$ °C) with a shaking speed of $120$ rpm, under a 12-h light–dark cycle with a light intensity of $21.0\pm 0.3$ μmol m^−2^ s^−1^. The stock culture was allowed to reach the stationary phase before inoculating the experimental cultures.

Control cultures were started by re-suspending biomass pellets obtained from the stock culture by rapid centrifugation ($4000\,\times \,g$ for 10 minutes) in fresh BG11 at an initial ${\text {OD}750} = 0.1$. The cultures were incubated in air at $30$ °C with a shaking speed of $120$ rpm, under a continuous cool white light with an intensity of $24.8 \pm 1.3$ μmol m^−2^ s^−1^ for the first 3 days, followed by a 12:12 light–dark cycle for the rest of the experiment. Faster growing PCC7942 cells were obtained by continuously diluting the culture. The periodic dilution with fresh media replenished nutrients in the culture and ensured high light penetration was maintained for faster growth. Continuously diluted cultures were started and incubated in the same way as the control but were periodically diluted to ${\text {OD}750}= 0.2$ with fresh BG11 medium once they grew to ${\hbox {OD}750} \ge 0.4$. This is the early exponential phase of growth (the range ${\hbox {OD}750}=$ 0.2–0.9 was observed to be the exponential phase of growth under the laboratory growth conditions applied).

All cultures were maintained in sterile conditions and axenic testing conducted on the stock culture prior to starting the experimental cultures.

### Analytical techniques

Analytical measurements were conducted during the same time window each day.

#### Measuring chlorophyll *a* content using UV spectroscopy

1 ml aliquot of culture was centrifuged in 1.5 ml Eppendorf tubes at 18,000 *rcf* for 10 min, after which 0.9 ml of the supernatant was removed. The pellet was resuspended in the remaining 0.1 ml supernatant and a volume of 0.9 ml 99.8% methanol was added. The solution was left in the dark for at least 15 min before centrifugation at 18,000 *rcf* for 5 min to obtain the chlorophyll extract (supernatant) that was analysed spectrophotometrically at 665 nm using a Thermo Scientific Evolution 201 UV–Visible spectrophotometer. The absorbance measurements were converted to dry mass concentration using a chlorophyll *a* extinction factor of $12.9447$ μg ml^−1^ cm A^−1^ [41].

#### Measuring cell concentration

Cell number (cells ml^−1^) was estimated from optical density (OD) measurements (turbidity) at $750$ nm using a Thermo Scientific Evolution 201 UV–Visible spectrophotometer. OD measurements are taken at $750$ nm since this is outside the range of absorbance of pigments (phycobilins, chlorophyll *a* and carotenoids) in the cyanobacterium. The ${\text {OD}750}$ readings were converted to cell number using standard curves calibrated by the authors over the growth cycle of the cyanobacterium. The spectrophotometer specific conversion factor was $2.95\cdot 10^{-9}$ ml cells^−1^ cm^−1^ A. Samples were diluted to ensure ${\hbox {OD}750} \le 0.8$ to remain in the linear region of the standard curves, where the linear correlation between cell number and ${\hbox {OD}750}$ hold, and then modified with the dilution factor to obtain the final concentrations. A Beckman Coulter Z2 Particle Counter was used to obtain cell number for calibration of the standard curve. All readings were adjusted with the background particle counts obtained using the pure diluent. In addition, the particle counter was used to obtain mean cell volumes.

#### Ferricyanide assay

Equation 1 is valid only when ferricyanide assays are conducted in mass transfer limited conditions. Ferricyanide reduction by *Synechocystis* sp. PCC6803 and *Chlorella vulgaris* is mass transfer limited at a ferricyanide concentration of $2.88$ mM for cell concentrations above $7.08\cdot 10^{7}$ cells ml^−1^ and above $2.4\cdot 10^{7}$cells ml^−1^, respectively [2, 28]. It is assumed that ferricyanide reduction by PCC7942 cells is similarly mass transfer limited at cell concentrations in the order $10^7$ cells ml^−1^. To account for the uncertainty in applying the same threshold to a different cyanobacterium species, a higher PCC7942 cell concentration of $6.78\cdot 10^{8}$ cells ml^−1^ and a lower ferricyanide concentration of 1 mM were used in this study to increase confidence that there were an excess of cells for a mass transport rather than a kinetically limited reaction.

Samples for ferricyanide assays were prepared by re-suspending biomass pellets obtained by rapid centrifugation from the culture of interest to $${\hbox {OD}750} = 2$$ (cell concentration of $$6.78\cdot 10^{8}$$) in modified BG11 media lacking iron and redox active compounds (ammonium iron citrate, citric acid, and $$\hbox {EDTNANa$_2$}$$). The medium was buffered to buffered to pH 7.0 using $$10\,{\text{mM}}$$ HEPES–NaOH as this has been reported to be the optimum pH for ferricyanide reduction by PCC7942 cells [19]. The samples were held in $$50$$ ml narrow mouth Erlenmeyer flasks and incubated at $$30$$ °C with a shaking speed of $$120$$ rpm under continuous light with an intensity of $$24.8 \pm 1.3$$ μmol m^−2^ s^−1^ throughout the assay. The shaking speed ensured all cells were fully suspended in the media. Complete suspension of the cells is a requirement for Eq. 4 to be applicable (see Calculations). Before ferricyanide solution was added, the samples were allowed to recover from the physical transfer and acclimatise to the new medium for 1 h, following which ferricyanide was added to the samples to a concentration of 1 mM, and a timer started. Aliquots of 1 ml were taken at the following times using a syringe: 0, 10, 30 and 120 min. The aliquots were filtered through a 0.45-μm syringe filters to remove cyanobacterium cells, into spectrophotometry cuvettes. Absorbance of the filtrate was measured using a Thermo Scientific Evolution 201 UV–Visible spectrophotometer. The absorbance readings were converted to molar concentration using an extinction coefficient of 1.05204 $$\text {mM}^{-1}$$ cm $$A^{-1}$$. Average $${[\hbox {Fe}(\hbox {CN})_{6}]^{3-}}$$ reduction rate over the 120 min period was calculated as the slope of the concentration vs. time graph.

### Confocal imagery and image processing

Aliquots of 0.1 ml were taken from cultures and left in the dark in 1.5-ml Eppendorf tubes for one hour to reduce cell division to a minimum [33]. Smaller aliquots of 10 μl were then taken from the dark-adapted cells and diluted to ${\text {OD}750} \le 0.5$ where necessary by adding fresh BG11 media to ensure that cells were sufficiently dispersed for confocal imaging and image processing. The 10 μl aliquots were sterilely transferred to autoclaved microscope slides and immobilised on BG11-agar medium. The BG11-agar media was prepared with the standard recipe, with the addition of 15 gL^−1^ of bacteriological agar as a solidifying agent.

A Leica TCS-SP5 microscope was used for laser-scanning confocal imaging. A 63$\times$ oil-immersion objective lens was used. Samples were excited at 633 nm with a HeNe laser at 50% power and autofluorescence recorded at 640–720 nm. The pinhole size was set to approximately 77 μm and 512 $\times$ 512 pixel images were captured at 400 Hz, averaging each scan frame 6 times to reduce noise while amplifying the fluorescence signal. The gain setting was set to ensure that images were captured without exceeding pixel saturation. For each sample, a minimum of 5–9 independent images were taken from different fields of view to have a statistically significant number of cells.

Images were processed using a custom-built MATLAB code. The algorithm binarises the images and then processes them using MATLAB’s *regionprops* function which superimposes an ellipse with the same normalised second central moment as the cell. The length and width of the cell were taken as the major and minor axis lengths of the ellipse, respectively. Pixel intensity thresholding and cut-off criteria, such as a minimum eccentricity or a maximum minor axis length, were used to avoid measuring length of cells which were out of focus, overlapped or dividing.

### Calculations

#### Estimation of mass transfer coefficient

The mass transfer coefficient for the transfer of ferricyanide from the bulk liquid to the surface of the cell membrane was estimated from published mass transfer correlations. For small microorganisms (characteristic length 1 $< d_{p}<$ 600 μm) suspended in an agitated system, Eq. 4 has been shown to be a good estimate of the mass transfer coefficient from the liquid phase to the microorganisms [42]:

4 $$\begin{aligned} k=\frac{2D_{{\text{AB}}}}{d_{{\text{p}}}}+0.31\cdot \left[ \frac{D_{{\text{AB}}}^{2}\mid \rho _{{\text{p}}}-\rho _{{\text{c}}}\mid g}{\mu _{{\text{c}}}}\right] ^{\frac{1}{3}} \end{aligned},$$

where *k* is the mass transfer coefficient, $d_{p}$ is the characteristic length of the particle (here, cell length, *L*), $D_{{\text{AB}}}$ is the diffusivity of A in B (here, of ferricyanide in cell medium), $\rho _{{\text{p}}}$ and $\rho _{{\text{c}}}$ are the particle (here, the cell) and continuous phase (here, BG11 medium) densities, respectively, ${\mu _{\mathrm{C}}}$ is the viscosity of the continuous phase, and *g* is the gravitational constant. The applicability of Eq. 4 to this study has been checked by reviewing the range of variables in the original experimental data used to fit the empirical equation [43]. Equation 4 was later confirmed to be valid for mass transfer to and from low-density particles with densities that differ only marginally from that of the continuous phase, such as microorganisms in media [44]. Importantly, the equation is fit using data from, and is valid for, free falling or free rising or suspended particles that are free to move under the influence of gravity in mixed vessels. As such, the second term on the right-hand side of Eq. 4 includes the gravitational term to take into account the free fall/rise of the microorganism by gravitational forces, while the first term on the right-hand side is due to molecular diffusion [42]. Researchers applying this method are encouraged to ensure the applicability of any mass transfer correlations used.

#### Stereological properties derived from projected dimensions

Stereological properties, namely geometric outer membrane surface area and cell volume, were evaluated using a rod-shaped model of PCC7942. In a rod-shaped model, the cells are assumed to be cylinders with hemispherical caps [45, 46]. The geometric outer membrane surface area (*A*) and cell volume (*V*) are then described by Eqs. 5 and 6, respectively, where *L* is cell length and *D* is the cell width [45, 46]. It should be noted that the actual surface area of the cells is likely to be higher due to folds on the cells’ membranes.

5 $$\begin{aligned} A= & {} \pi D(L-D)+\pi D^{2}=\pi DL \end{aligned}$$

6 $$\begin{aligned} V= & {} 1/4\cdot \pi D^{2}(L-D)+1/6\cdot \pi D^{3}=1/4\cdot \pi D^{2}(L-1/3\cdot D) \end{aligned}$$

In cases where ferricyanide is thought to react at the plasma membrane, measured *L* and *D* values should be corrected to take into account the thickness of the periplasmic space. This can be done by subtracting 2$\times$ the thickness of the periplasmic space from each dimension. For *Synechococcus* sp., the thickness of the periplasmic space can be assumed to be roughly equivalent to the thickness of the peptidoglycan layer. The thickness of the peptidoglycan layer is 10 nm [47].

#### Statistical analysis

Both the control and continuously diluted cultures were grown in three independent replicates. Ferricyanide assays and confocal imaging were conducted for each replicate and means taken. For confocal image processing, the weighted mean length and width of cells were calculated to take into account the different number of cells in each image. Student’s *t* tests at 5% significance levels were performed for statistical analysis.

## Supplementary information

**Additional file 1.** File containing the following additional information discussed in the main text: growth curves, chlorophyll* a* and pH profiles, sample raw confocal images, sample processed confocal image, cell length histograms, theoretical iron concentration profile, correlations between ([Fe^3+^]_b_ − [Fe^3+^]_s_) and morphological parameters and iron concentration, and a comparison of Coulter counter volume measurements and rod-shaped model evaluation of volume.

## Data Availability

The datasets during and/or analysed during the current study available from the corresponding author on reasonable request.
