# Supplementary material for: Quantitative analysis of the effects of morphological changes on extracellular electron transfer rates in cyanobacteria
Source: Biotechnol Biofuels. 2020 Aug 26;13:150. doi: 10.1186/s13068-020-01788-8 (PMC7449014; doi:10.1186/s13068-020-01788-8)
Supplement: Supplementary file 1 — Additional file 1. File containing the following additional information discussed in the main text: growth curves, chlorophyll a and pH profiles, sample raw confocal images, sample processed confocal image, cell length histograms, theoretical iron concentration profile, correlations between ([Fe3+]b − [Fe3+]s) and morphological parameters and iron concentration, and a comparison of Coulter counter volume measurements and rod-shaped model evaluation of volume. [file 13068_2020_1788_MOESM1_ESM.pdf]

Supplementary information: Quantitative analysis of the effects of morphological changes on extracellular electron transfer rates in cyanobacteria

Tonny I. Okedi, Adrian C. Fisher, Kamran Yunus

List of Figures

S.1 Culture growth curves. 1

S.2 Sample raw autofluorescence confocal images of PCC7942 cells at 63x magnification on days 1 and 5. 2

S.3 Sample raw autofluorescence confocal images of PCC7942 cells at 63x magnification on days 9 and 13. 3

S.4 Sample raw autofluorescence confocal images of PCC7942 cells at 63x magnification on days 17 and 21. 4

S.5 Sample confocal image 5

S.6 Cell length histograms 6

S.7 Ferricyanide reduction rates normalised by chlorophyll. 7

S.8 Chlorophyll a content profiles 8

S.9 Correlations between $\left([Fe^{3+}]_b - [Fe^{3+}]s\right)$ and cell morphologic properties 9

S.10 Estimated iron concentration profiles 12

S.11 Comparison of cell volume from coulter counter measurements and rod-shaped model 13

S.12 Culture pH profiles 14

Growth curves

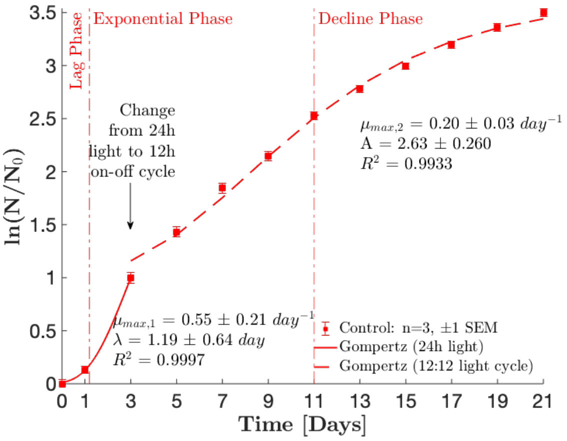

(a)

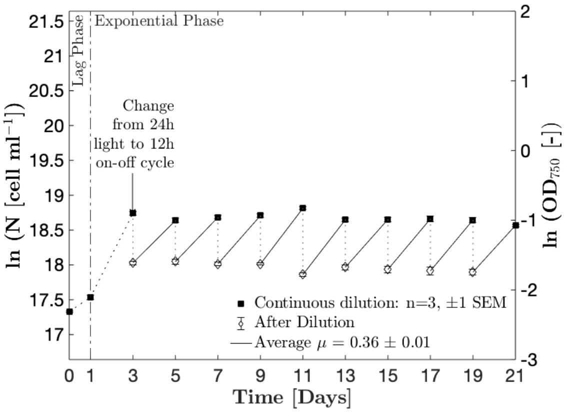

(b)

Figure S.1: **Culture growth curves. (a)** Specific growth rates (μ_{max}), lag times (λ) and maximal log of the relative cell number (A) were estimated by fitting the Gompertz model to the cell concentration profile [1]. Due to the change in light regime from 24h continuous light to a 12:12 light-dark cycle on day 3, the Gompertz model was fit twice. Since the Gompertz model requires at least 4 points to fit, it was fit first from day 3 to the end of the experiment to obtain $\mu_{max,2}$ and A. The fitted value of A was then fixed and the Gompertz model fit a second time from day 0 to day 3 to obtain λ and $\mu_{max,1}$. Each parameter is quoted with ±95% confidence interval. **(b)** For the continuously diluted cultures that were maintained in exponential growth, average growth rate (μ) was estimated from the gradients of the solid lines. Y-axis error bars for $ln(N/N_0)$ data points show ±1 Standard Error of the Mean (SEM). Where no error bars are visible, they are smaller than the markers.

Fig. S.1a shows a large error in the estimation of the lag time. This is due to the relatively low frequency of measurements (every 48 hours except on day 1 where measurements were taken 24 hours after inoculation). The confidence bounds of the lag time may be narrowed by increasing the frequency of cell concentration measurements. The size of the incubator restricted the volume of flasks that could be used for culturing the cells in 3 replicates for each growth regime. In turn, this limited the volume of culture, since the depth of the culture had be shallow enough to allow sufficient light penetration. Due to the significant withdrawal of culture volume for measurements, a trade-off was made between frequency of readings, and length of the experiment. In order to have the experiment run for at least 20 days, it was decided to conduct ferricyanide assays every 4 days, and to measure the other metrics (cell concentration, pH and chlorophyll a) every 48 hours. In addition, significant withdrawal of the culture volume would have altered the natural growth curve, particularly for the control cultures, as it changes the light penetration appreciably.

Sample Confocal Images

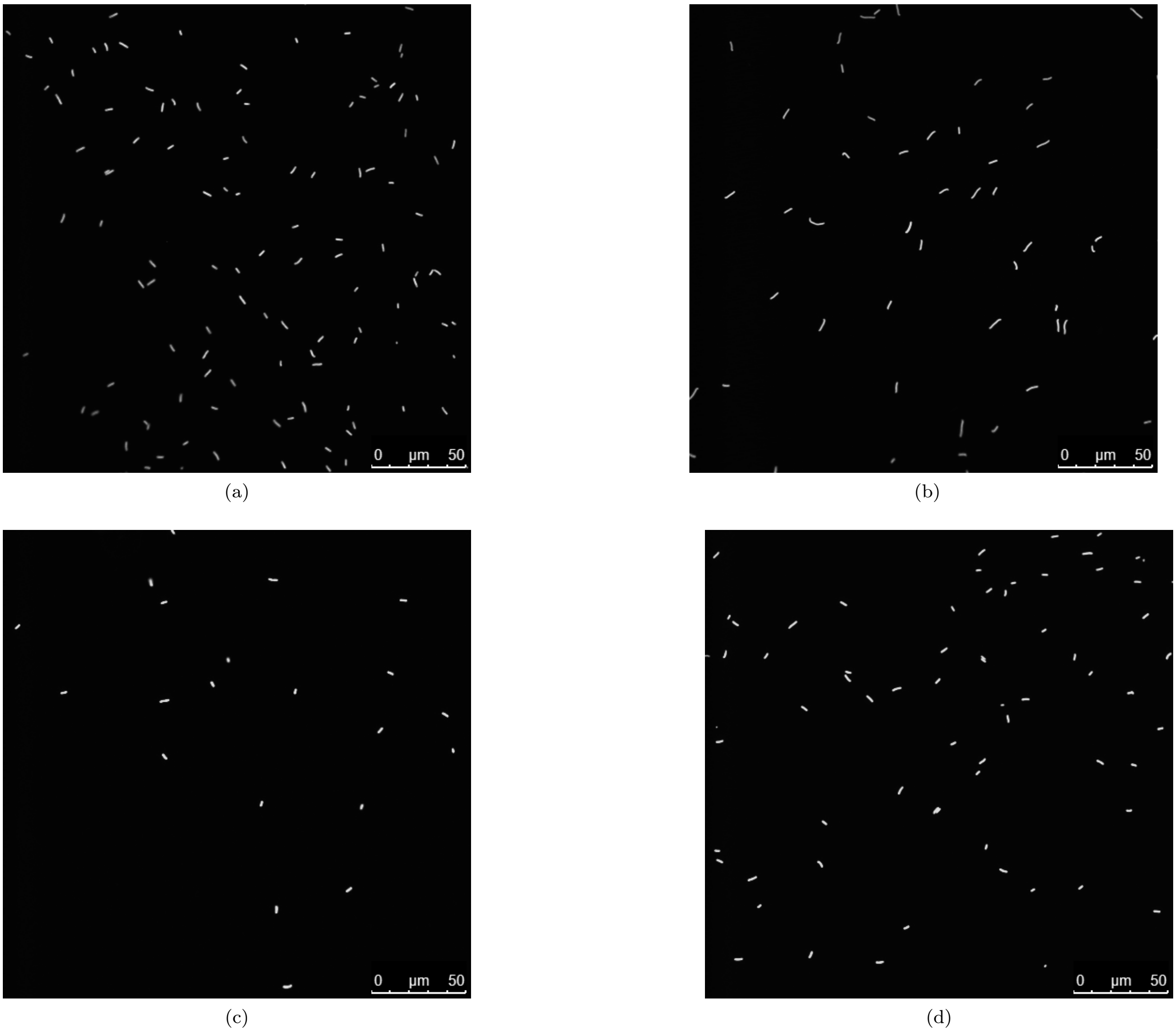

Figure S.2: **Sample raw autofluorescence confocal images of PCC7942 cells at 63x magnification on days 1 and 5.** **(a)** Continuously diluted cells day 1 (24 hours after inoculation). **(c)** Continuously diluted cells day 5. **(b)** Control cells day 1 (24 hours after inoculation). **(d)** Control cells day 5. The cultures were in the lag phase for the first 22-24 hours after inoculation. Cells are seen to be visibly shorter on day 5 when the cultures are in the exponential phase.

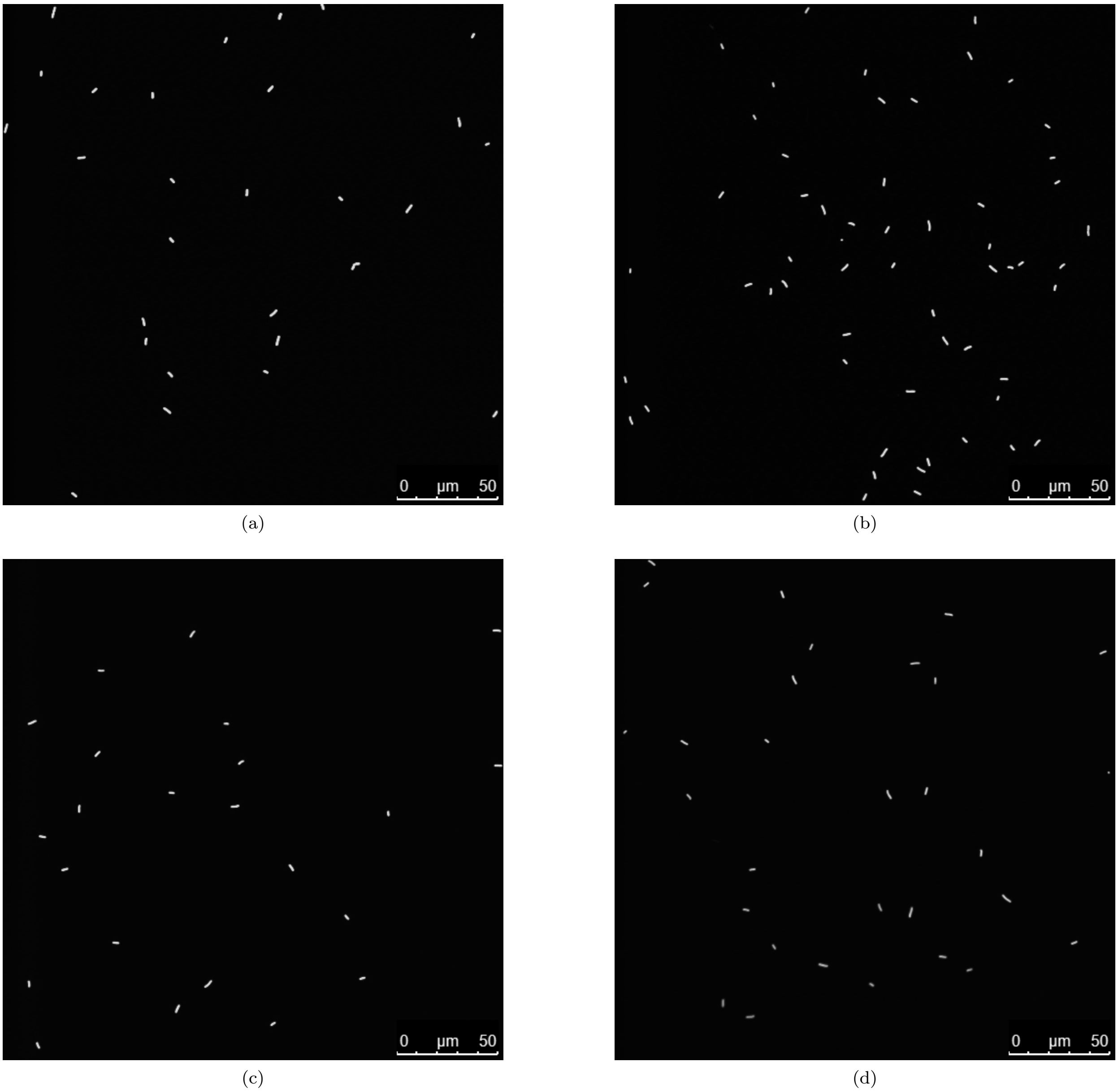

Figure S.3: **Sample raw autofluorescence confocal images of PCC7942 cells at 63x magnification on days 9 and 13. (a)** Continuously diluted cells day 9. **(c)** Continuously diluted cells day 13. **(b)** Control cells day 9. **(d)** Control cells day 13. Control cells were in the decline phase from day 11 however, no visible difference in cell length compared to continuously diluted cells was seen by day 13.

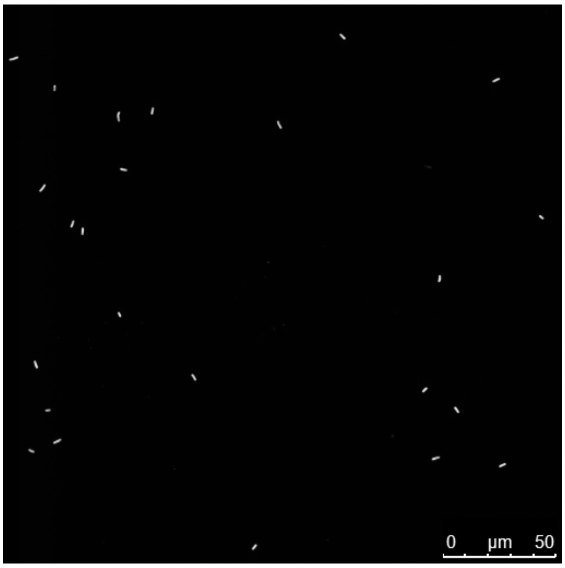

(a)

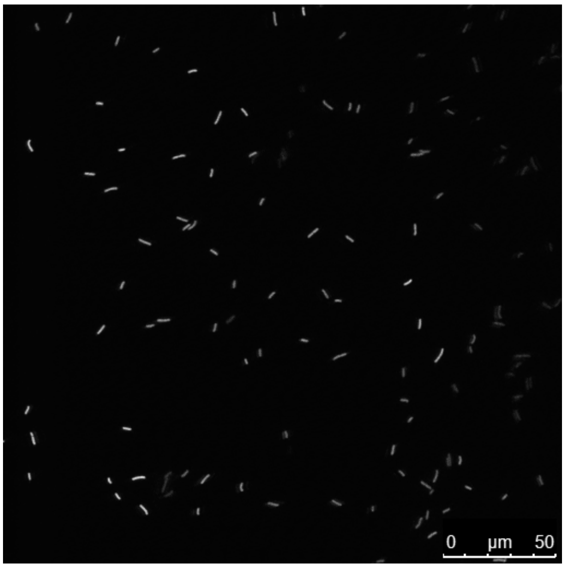

(b)

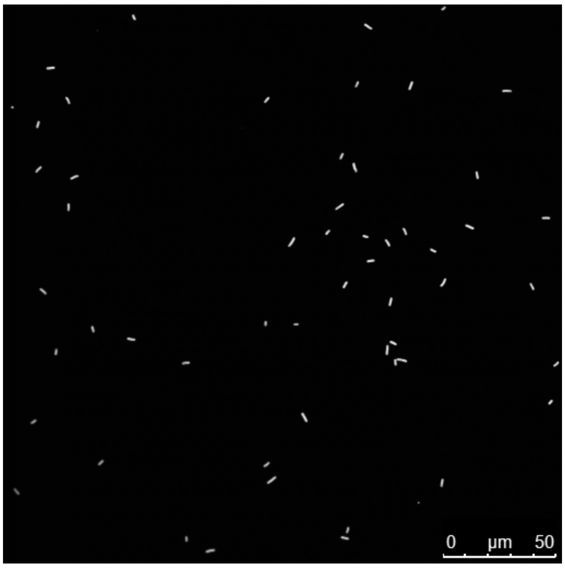

(c)

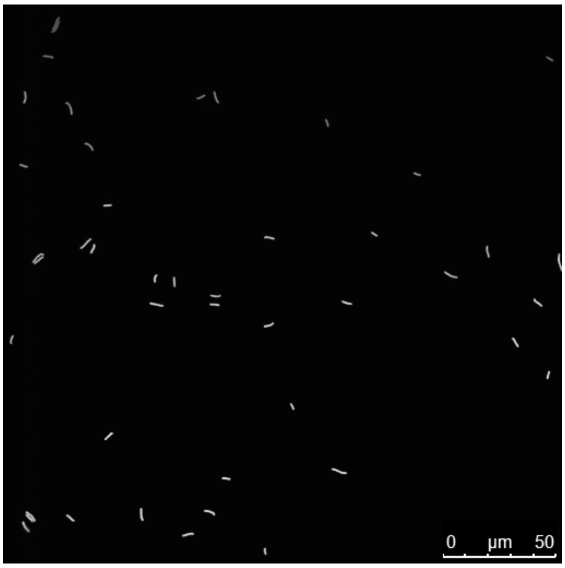

(d)

Figure S.4: **Sample raw autofluorescence confocal images of PCC7942 cells at 63x magnification on days 17 and 21.** **(a)** Continuously diluted cells day 17. **(c)** Continuously diluted cells day 21. **(b)** Control cells day 17. **(d)** Control cells day 21. Control cells were now firmly in the decline phase and are visibly longer than the continuously diluted cells on days 17 and 21.

Sample processed image

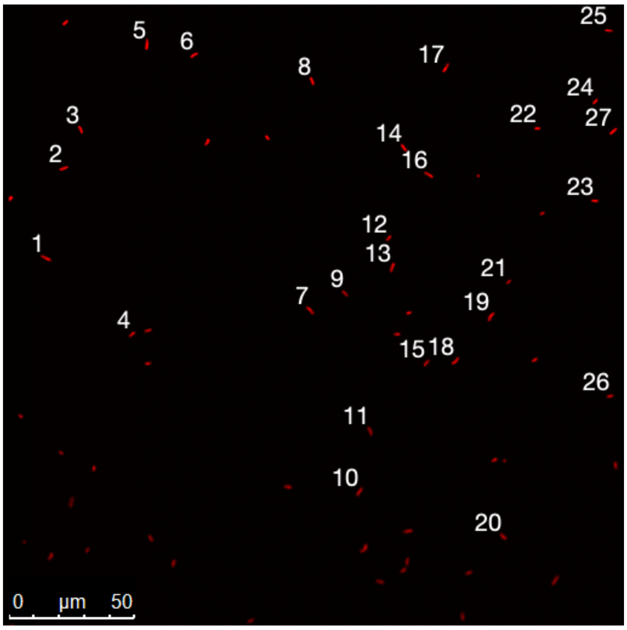

(a)

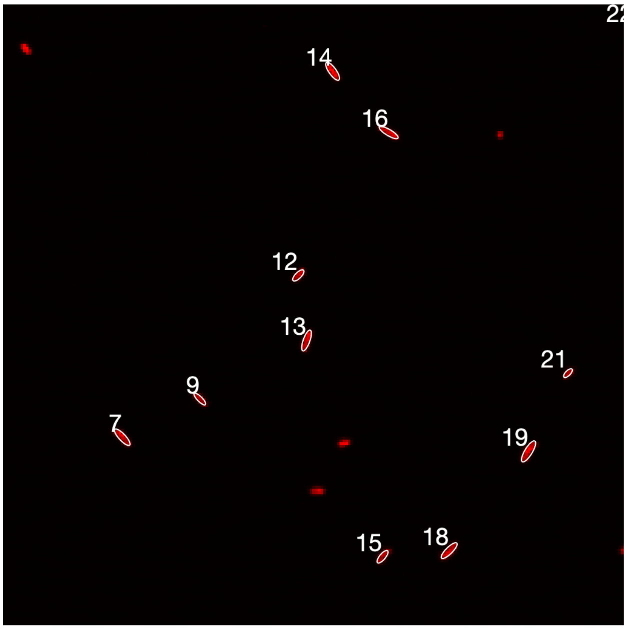

(b)

Figure S.5: **Sample processed confocal image. (a)** Autofluorescence confocal image of PCC7942 cells at 63x magnification. Numbered cells are those in focus, with sufficient contrast for further image processing using MATLAB. **(b)** Zoomed in section of (a) following image processing, showing ellipses superimposed on the cells. The major and minor axis lengths of the superimposed ellipse was taken to be the length and width of a cell respectively.

Cell size histograms

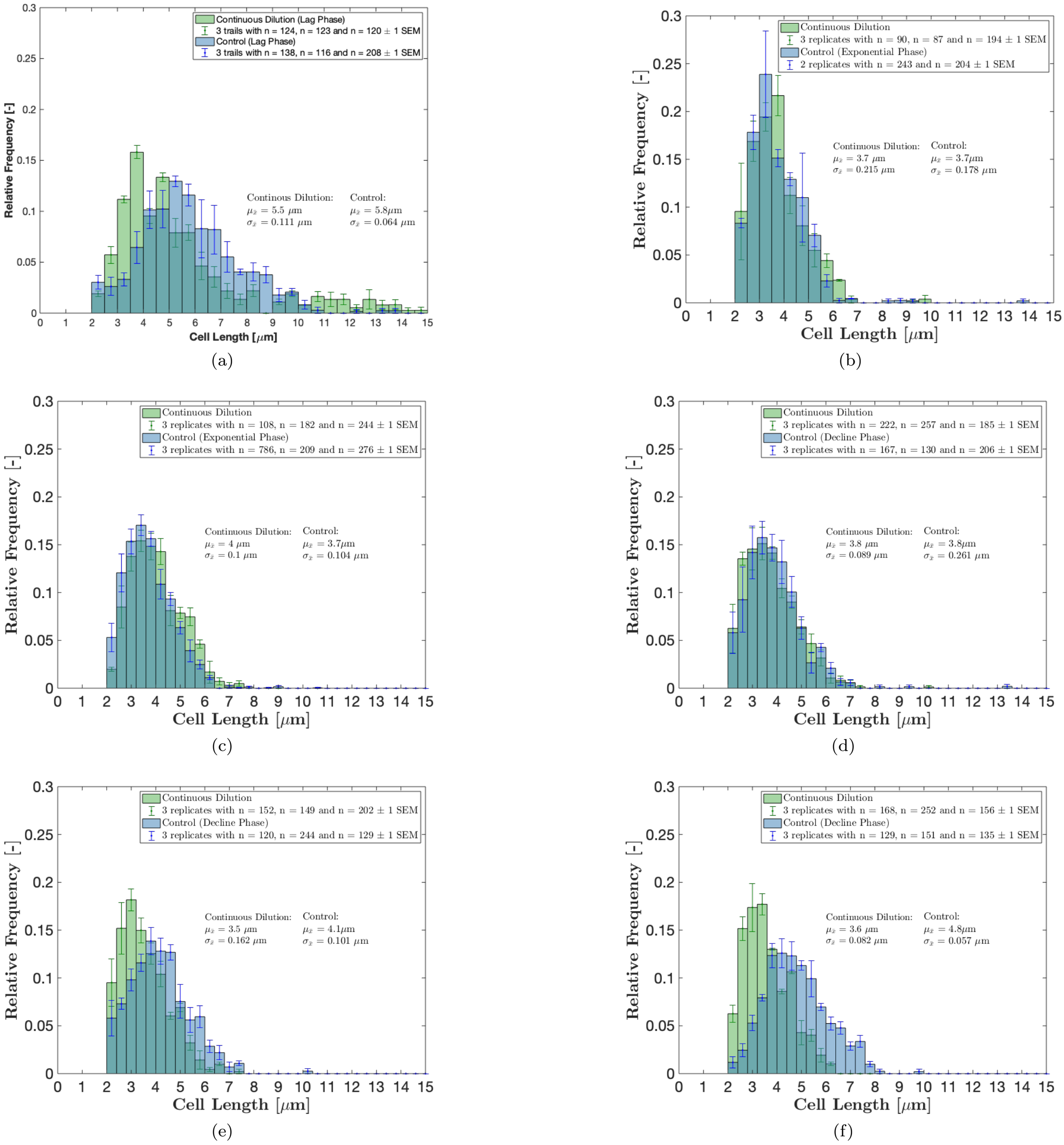

Figure S.6: **Cell length histograms. (a)** day 1 **(b)** day 5, **(c)** day 9, **(d)** day 13, **(e)** day 17 and **(f)** day 21. Cells in the exponential phase (control cells on days 5,7, 9, and continuously diluted cells on days 5,7,9,13,17,21) have a right tailed histogram. As the control cells enter into the decline phase and transition to the stationary phase, the distribution of cell sizes became more normally distributed.

Ferricyanide reduction rates normalised by chlorophyll *a* content

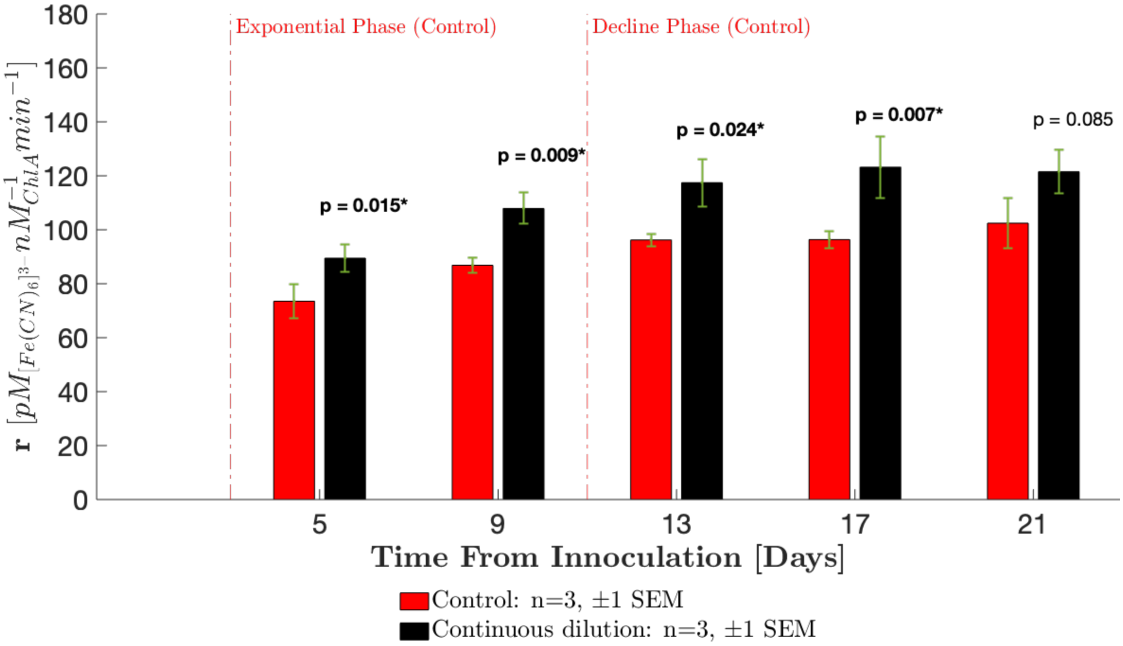

Figure S.7: **Ferricyanide reduction rates normalised by chlorophyll.** Reduction rates normalised by chlorophyll *a* content are provided for comparison with PCC7942 studies found in literature. Error bars show ±1 SEM. To assess significance of the pairwise difference in rates, a one-tailed Student's t-test at 5% significance level was conducted, with alternative hypothesis that reduction rates are higher for the continuously diluted cultures. Significant p-values are asterisked (above bars).

Chlorophyll *a* profile

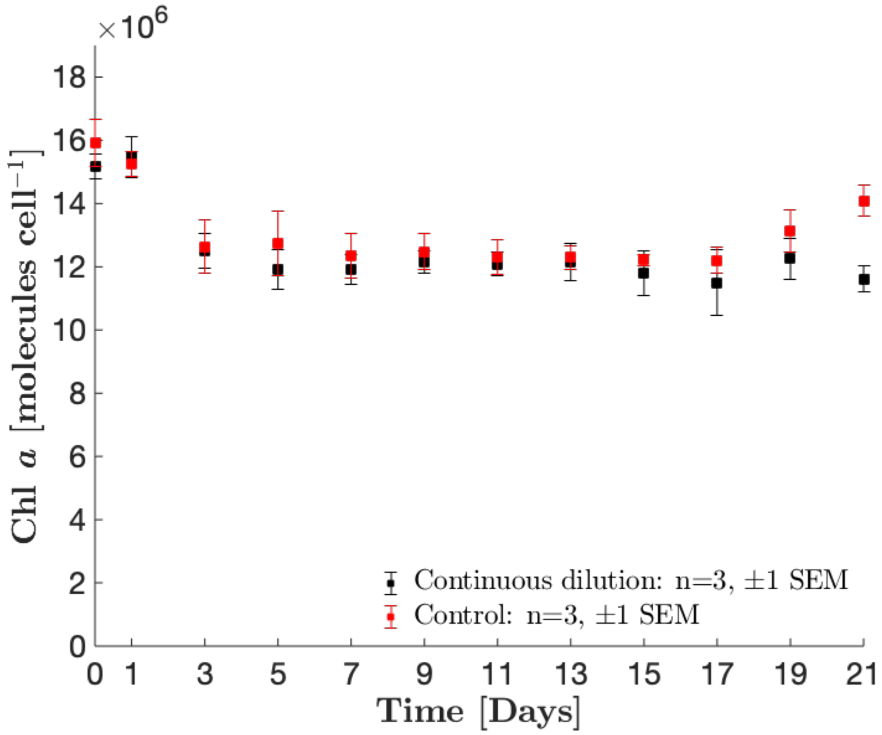

Figure S.8: **Chlorophyll *a* content profiles.** Chlorophyll *a* content was measured as an indicator of the effects of the different growth conditions on the photosystems. Chlorophyll *a* content remained constant at around $12.5 \cdot 10^6$ *molecules cell*$^{-1}$ for both cultures over the majority of the study period. A marginal increase in chlorophyll *a* content to $14.0 \cdot 10^6$ *molecules cell*$^{-1}$ was observed for the control cultures from day 17 onwards, likely due to reduced light penetration as cell density increased. Error bars show ±1 SEM. Where no error bars are visible, they are smaller than the markers.

Correlations

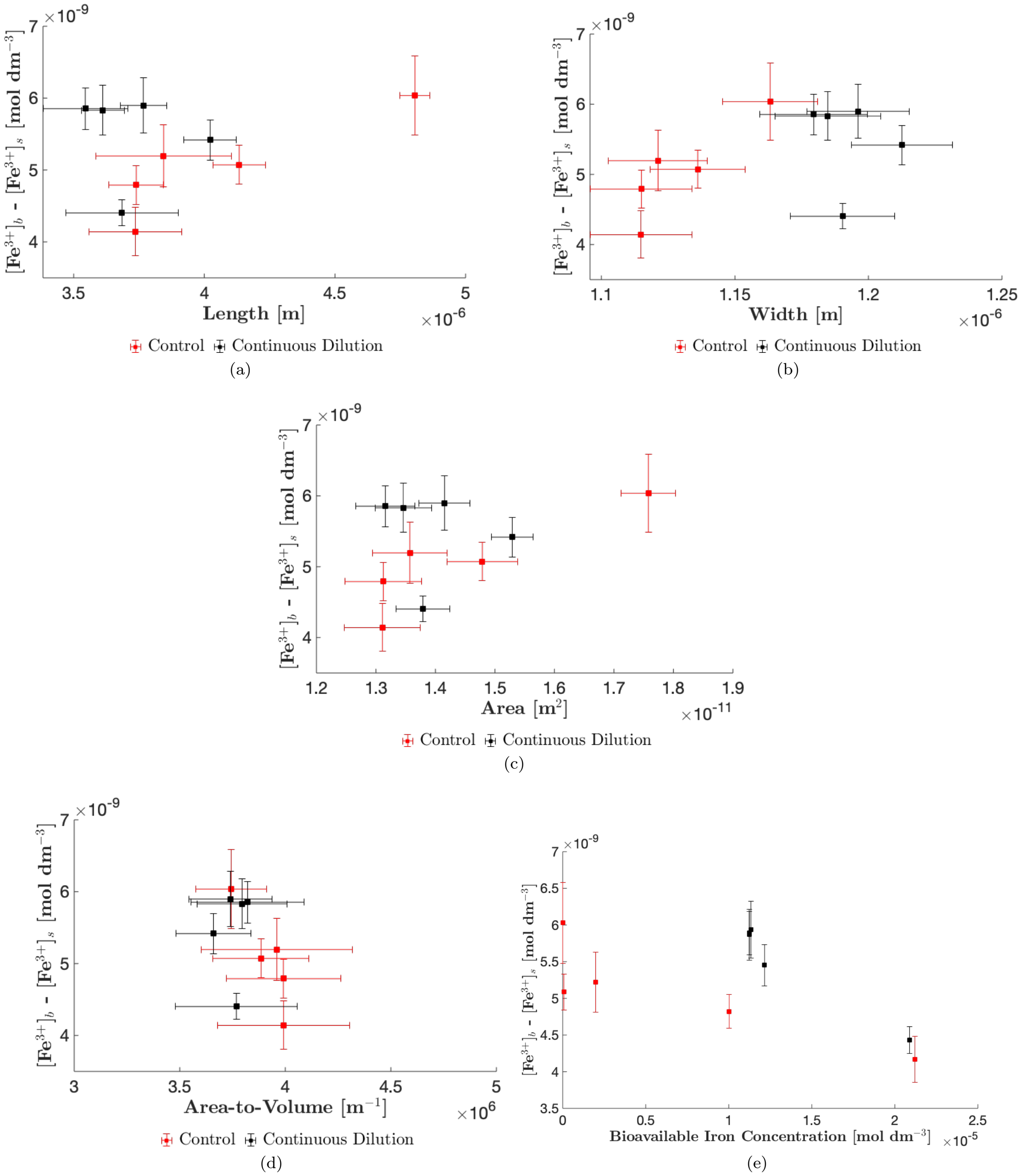

Figure S.9: Correlations between $\left([Fe^{3+}]_b - [Fe^{3+}]s\right)$ and **(a)** cell length, **(b)** cell width, **(c)** cell geometric surface area **(d)** cell surface area-to-volume ratio and **(e)** BG11 iron concentration. No strong linear correlations are seen with any of the dimensional or stereological properties. A strong negative correlation with BG11 iron concentration is seen for both cultures.

Estimated BG11 iron concentration profiles

Simplified model for iron concentration profiles

The bioavailable iron in BG11 is from ammonium ferric citrate[2]. The evolution of the bioavailable iron concentration in the media can be estimated using a mass balance equation and a model for the growth kinetics of the culture. The rate of change of iron in the BG11 media is equivalent to the negative rate of consumption of iron by the cells, eq. S.1,

$$\frac{d[Fe]_{bio}}{dt} = -r \tag{S.1}$$

where $[Fe]_{bio}$ is the concentration of bioavailable iron in the BG11 media in $mol\ dm^{-3}$, r is the iron uptake rate by cyanobacteria in $mol\ dm^{-3}\ day^{-1}$, and t is time in $days$. The rate of of consumption of dissolved inorganic iron or iron-siderephore complexes by cyanobacteria can be calculated from the uptake rate constant, k_{in} (eq. S.2) as defined in [3].

$$k_{in} = \frac{r}{[Fe]_{bio}} \tag{S.2}$$

where k_{in} has the units of $dm^3\ cell^{-1}\ day^{-1}$. The uptake rate constant is linearly correlated to the area of the cyanobacteria [3]. The cyanobacteria *Synechococcus elongatus sp.* PCC7942, is a siderophore producer. In order to simplify calculations, it is assumed that the cells uptake iron primarily through siderophore secretion, and negligible amounts through a reductive mechanism. The value of k_{in} in units of $dm^3\ cell^{-1}\ hr^{-1}$ for iron-siderophore complexes can then be calculated using eq. S.3, which is a linear fit of empirical data [3]:

$$k_{in} = 6.27 \cdot 10^{-15} * A \tag{S.3}$$

where A is the surface area of the cyanobacterium in μm^2 . Thus, by combining eq. S.1, S.2 and S.3, the rate of change of iron in the BG11 media per day can be written as:

$$\frac{d[Fe]_{bio}}{dt} = -6.27 \cdot 10^{-15} * 24 * A * N * [Fe]_{bio} \tag{S.4}$$

where N is the cell number in $cells\ dm^{-3}$. In the continuously diluted cultures, it was shown in Fig. 3 in the main text that A remains constant during the exponential phase of growth. In order to simplify calculations for the control culture, it is assumed that the value of A also remains constant at its average value over the duration of the experiment.

The growth kinetics for the continuously diluted cultures are modelled using the standard exponential growth equation, eq. S.5. It was assumed that the equation also applies in the lag phase since there are no equations

describing the rate of change of cells during this period. This simplification over estimates the cell number during the first 24 hours of growth, and as a consequence, the value of $d[Fe]_{bio}/dt$. However, because there is evidence that the majority of iron uptake occurs in the lag phase, it is reasonable to have a higher uptake rate during this time[4, 5]:

$$\frac{dN}{dt} = \mu * N \tag{S.5}$$

where μ is the average growth rate over the 21 day experiment as shown in Fig. S.1b. The growth kinetics of the control cultures are modelled using the Gompertz model[1]:

$$y = ln\left(\frac{N}{N_0}\right) = Ae^{-\left(e^{(e\cdot\mu_{max}\cdot(\lambda-t))/A+1}\right)} \tag{S.6}$$

Thus, the rate of change of $y = ln(N/N_0)$ is given by eq. S.7:

$$\frac{dy}{dt} = \mu_{max}\cdot exp\left(\frac{e\cdot\mu_{max}\cdot(\lambda-t)}{A} - e^{(e\cdot\mu_{max}\cdot(\lambda-t))/A+1} + 2\right) \tag{S.7}$$

where μ_{max}, λ and A are as previously defined and fitted in Fig. S.1a. Equations S.4 and S.5 or S.7 for the continuously diluted or control cultures respectively were solved simultaneously using MATLAB's *ode23s* solver. For the control culture, the initial cell number, N_0, was the cell number at inoculation, ($\approx 3.39\cdot10^4$ $cells\ dm^{-3}$), and the initial concentration of bioavailable iron in the media, $[Fe]_{bio,0}$, was the concentration of iron in fresh BG11 media ($\approx$1.3 $mg\ dm^{-3}$ or $2.29\cdot10^{-5}$ $mol\ dm^{-3}$).

Initial conditions for the continuously diluted cultures

Since the continuously diluted cultures were diluted every 48 hours to $OD_{750} = 2$, starting two days after entering the exponential phase of growth (day 3), equations S.4 and S.5 above were solved for the first 72 hours, and then for every 48 hour period that follows a dilution. For the first 72 hours, $[Fe]_{bio,0}$ was the concentration of iron in fresh BG11 media. At the start of dilutions, $[Fe]^t_{bio,0}$ was corrected using a mass balance ($Accumulation = Initial - Output + Input$) to take into account the dilution step. During dilution, half of the culture (by volume) was removed and replaced with an equal volume of fresh BG11 media. Therefore, the amount of iron in the media immediately after a dilution event is given by the following mass balance:

$$[Fe]^t_{bio,0} * V = [Fe]^{t-1}_{bio} * V - \frac{1}{2} * [Fe]^{t-1}_{bio} * V + \frac{1}{2} * [Fe]_{BG11} * V \tag{S.8}$$

where V is the culture volume in dm^3, $[Fe]^{t-1}_{bio,0}$ is the bioavailable iron concentration at the end of the previous 48 hour period and $[Fe]_{BG11}$ is the bioavailable iron concentration in fresh BG11 media. Equation S.8 can be simplified to eq. S.9 by dividing both sides by V:

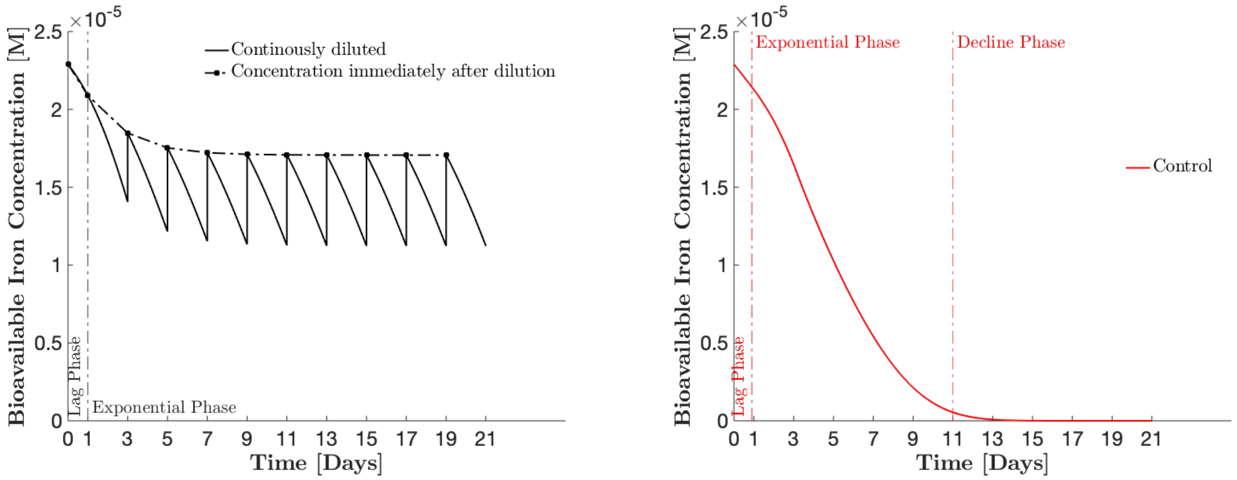

(a) (b)

Figure S.10: **(a)** Estimated bioavailable iron concentration profile for continuously diluted cultures. **(b)** Estimated bioavailable iron concentration profile for control cultures.

$$[Fe]^{t}_{bio,0} = [Fe]^{t-1}_{bio} - \frac{1}{2} * [Fe]^{t-1}_{bio} + \frac{1}{2} * [Fe]_{BG11} \tag{S.9}$$

The initial cell number, N_0, was the cell number at inoculation of the culture for the first 72 hour period, ($\approx 3.39{\cdot}10^4$ $cells\ dm^{-3}$). For the subsequent 48 hour periods, N_0 was the cell number immediately after dilution to $OD_{750} = 0.2$ ($\approx 6.78{\cdot}10^4$ $cells\ dm^{-3}$).

Estimated iron concentration profiles

Figure S.10 shows the calculated iron concentration profiles. In the continuously diluted cultures, the iron concentration immediately after a dilution reaches its terminal value by day 13 and does not change further for the remainder of the experiment. The iron concentration remains constricted within a narrow window, declining from $\approx 1.71{\cdot}10^{-5}$ $mol\ dm^{-3}$ immediately after dilution to $\approx 1.21{\cdot}10^{-5}$ after 48 hours. Interestingly, the maximum ferricyanide reduction rate was also reached on day 13, suggesting a link between iron assimilation and extracellular electron transfer[6].

The iron concentration in the control cultures decreases and reaches zero by day 13. At the beginning of the decline phase (day 11), the iron concentration relative to the iron concentration in fresh BG11 media is 0.02. This value is the same as that measured by Watanabe et. al after culturing *Synechococcus elongatus sp.* PCC7942 for 7 days, at which time the culture was in stationary phase [5]. In their study, the culture was grown in continuous light, under 2% CO_2, and a light intensity of 40 $\mu mol\ m^{-2}s^{-1}$, resulting in μ_{max} of 1.2 day^{-1} (estimated from the growth curve reported) or 2.5 times faster than in this work.

Comparison to particle sizer measurements

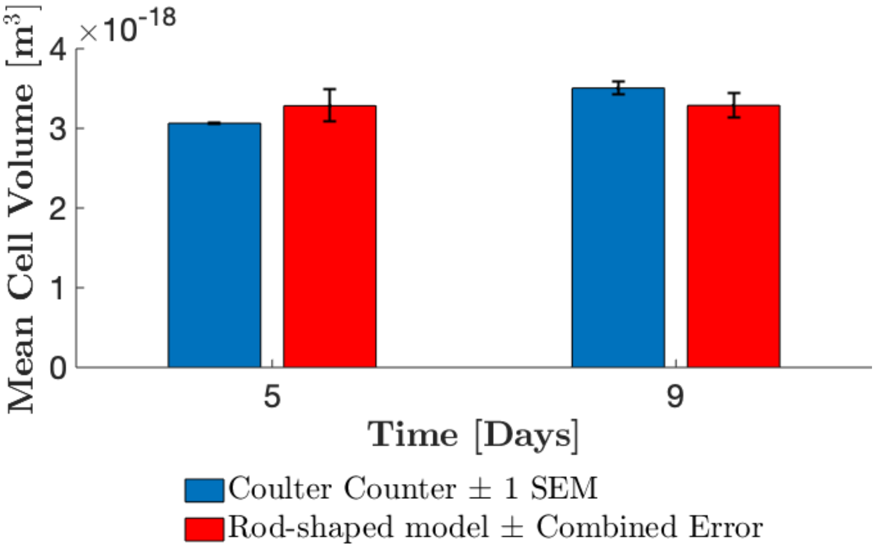

Figure S.11: Mean cell volume of *Synechococcus sp.* PCC7942 cells in the control culture on days 5 and 9 obtained using a coulter counter (blue bars) and evaluated using the rod-shaped model (red bars). Combined error in the volume value evaluated using the rod-shaped model (ΔV) was calculated using error propagation: $(\Delta V/V)^2 = 2 \cdot (\Delta D/D)^2 + (\Delta L/L)^2 + 3 \cdot (\Delta D/D)^2$. Volumes on both days agreed within experimental uncertainty, justifying the use of the model to calculate stereological properties of the cells from projected cell dimensions obtained using confocal microscopy.

pH profile

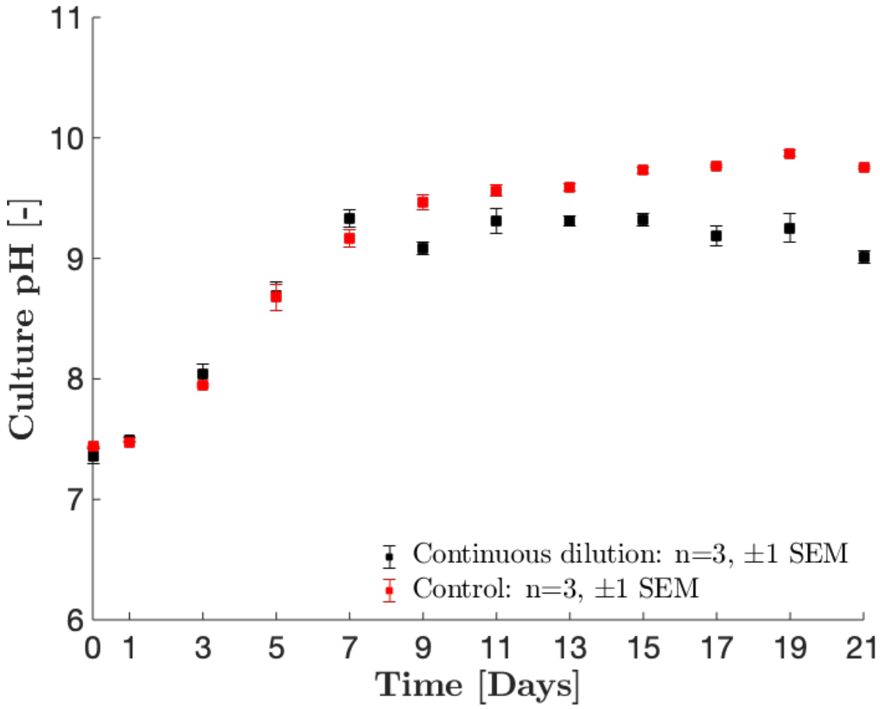

Figure S.12: **Culture pH profiles.** Evolution of culture pH was also measured as an indicator of the changing external environment of the cells. Cyanobacteria are alkaliphilic and increase the pH of their environment by assimilating carbonate ions and compounds during photosynthesis [7, 8].The pH of both cultures increased from 7.4 to approximately 9.3 over a period of 11 days. After this, control cultures experienced a slight increase in pH to approximately 9.7, which may be due to increased number of cells consuming more carbonate ions and compounds. Error bars show ±1 SEM. Where error bars are not visible, they are smaller than the marker size.
